# Search for heavy *ZZ* resonances in the $\ell ^+\ell ^-\ell ^+\ell ^-$ and $\ell ^+\ell ^-\nu \bar{\nu }$ final states using proton–proton collisions at $\sqrt{s}= 13$ $\text {TeV}$ with the ATLAS detector

**DOI:** 10.1140/epjc/s10052-018-5686-3

**Published:** 2018-04-11

**Authors:** M. Aaboud, G. Aad, B. Abbott, O. Abdinov, B. Abeloos, S. H. Abidi, O. S. AbouZeid, N. L. Abraham, H. Abramowicz, H. Abreu, R. Abreu, Y. Abulaiti, B. S. Acharya, S. Adachi, L. Adamczyk, J. Adelman, M. Adersberger, T. Adye, A. A. Affolder, Y. Afik, T. Agatonovic-Jovin, C. Agheorghiesei, J. A. Aguilar-Saavedra, S. P. Ahlen, F. Ahmadov, G. Aielli, S. Akatsuka, H. Akerstedt, T. P. A. Åkesson, E. Akilli, A. V. Akimov, G. L. Alberghi, J. Albert, P. Albicocco, M. J. Alconada Verzini, S. C. Alderweireldt, M. Aleksa, I. N. Aleksandrov, C. Alexa, G. Alexander, T. Alexopoulos, M. Alhroob, B. Ali, M. Aliev, G. Alimonti, J. Alison, S. P. Alkire, B. M. M. Allbrooke, B. W. Allen, P. P. Allport, A. Aloisio, A. Alonso, F. Alonso, C. Alpigiani, A. A. Alshehri, M. I. Alstaty, B. Alvarez Gonzalez, D. Álvarez Piqueras, M. G. Alviggi, B. T. Amadio, Y. Amaral Coutinho, C. Amelung, D. Amidei, S. P. Amor Dos Santos, S. Amoroso, C. Anastopoulos, L. S. Ancu, N. Andari, T. Andeen, C. F. Anders, J. K. Anders, K. J. Anderson, A. Andreazza, V. Andrei, S. Angelidakis, I. Angelozzi, A. Angerami, A. V. Anisenkov, N. Anjos, A. Annovi, C. Antel, M. Antonelli, A. Antonov, D. J. Antrim, F. Anulli, M. Aoki, L. Aperio Bella, G. Arabidze, Y. Arai, J. P. Araque, V. Araujo Ferraz, A. T. H. Arce, R. E. Ardell, F. A. Arduh, J-F. Arguin, S. Argyropoulos, M. Arik, A. J. Armbruster, L. J. Armitage, O. Arnaez, H. Arnold, M. Arratia, O. Arslan, A. Artamonov, G. Artoni, S. Artz, S. Asai, N. Asbah, A. Ashkenazi, L. Asquith, K. Assamagan, R. Astalos, M. Atkinson, N. B. Atlay, K. Augsten, G. Avolio, B. Axen, M. K. Ayoub, G. Azuelos, A. E. Baas, M. J. Baca, H. Bachacou, K. Bachas, M. Backes, P. Bagnaia, M. Bahmani, H. Bahrasemani, J. T. Baines, M. Bajic, O. K. Baker, P. J. Bakker, E. M. Baldin, P. Balek, F. Balli, W. K. Balunas, E. Banas, A. Bandyopadhyay, Sw. Banerjee, A. A. E. Bannoura, L. Barak, E. L. Barberio, D. Barberis, M. Barbero, T. Barillari, M-S Barisits, J. T. Barkeloo, T. Barklow, N. Barlow, S. L. Barnes, B. M. Barnett, R. M. Barnett, Z. Barnovska-Blenessy, A. Baroncelli, G. Barone, A. J. Barr, L. Barranco Navarro, F. Barreiro, J. Barreiro Guimarães da Costa, R. Bartoldus, A. E. Barton, P. Bartos, A. Basalaev, A. Bassalat, R. L. Bates, S. J. Batista, J. R. Batley, M. Battaglia, M. Bauce, F. Bauer, H. S. Bawa, J. B. Beacham, M. D. Beattie, T. Beau, P. H. Beauchemin, P. Bechtle, H. P. Beck, H. C. Beck, K. Becker, M. Becker, C. Becot, A. J. Beddall, A. Beddall, V. A. Bednyakov, M. Bedognetti, C. P. Bee, T. A. Beermann, M. Begalli, M. Begel, J. K. Behr, A. S. Bell, G. Bella, L. Bellagamba, A. Bellerive, M. Bellomo, K. Belotskiy, O. Beltramello, N. L. Belyaev, O. Benary, D. Benchekroun, M. Bender, N. Benekos, Y. Benhammou, E. Benhar Noccioli, J. Benitez, D. P. Benjamin, M. Benoit, J. R. Bensinger, S. Bentvelsen, L. Beresford, M. Beretta, D. Berge, E. Bergeaas Kuutmann, N. Berger, L. J. Bergsten, J. Beringer, S. Berlendis, N. R. Bernard, G. Bernardi, C. Bernius, F. U. Bernlochner, T. Berry, P. Berta, C. Bertella, G. Bertoli, I. A. Bertram, C. Bertsche, G. J. Besjes, O. Bessidskaia Bylund, M. Bessner, N. Besson, A. Bethani, S. Bethke, A. Betti, A. J. Bevan, J. Beyer, R. M. Bianchi, O. Biebel, D. Biedermann, R. Bielski, K. Bierwagen, N. V. Biesuz, M. Biglietti, T. R. V. Billoud, H. Bilokon, M. Bindi, A. Bingul, C. Bini, S. Biondi, T. Bisanz, C. Bittrich, D. M. Bjergaard, J. E. Black, K. M. Black, R. E. Blair, T. Blazek, I. Bloch, C. Blocker, A. Blue, U. Blumenschein, Dr. Blunier, G. J. Bobbink, V. S. Bobrovnikov, S. S. Bocchetta, A. Bocci, C. Bock, M. Boehler, D. Boerner, D. Bogavac, A. G. Bogdanchikov, C. Bohm, V. Boisvert, P. Bokan, T. Bold, A. S. Boldyrev, A. E. Bolz, M. Bomben, M. Bona, M. Boonekamp, A. Borisov, G. Borissov, J. Bortfeldt, D. Bortoletto, V. Bortolotto, D. Boscherini, M. Bosman, J. D. Bossio Sola, J. Boudreau, E. V. Bouhova-Thacker, D. Boumediene, C. Bourdarios, S. K. Boutle, A. Boveia, J. Boyd, I. R. Boyko, A. J. Bozson, J. Bracinik, A. Brandt, G. Brandt, O. Brandt, F. Braren, U. Bratzler, B. Brau, J. E. Brau, W. D. Breaden Madden, K. Brendlinger, A. J. Brennan, L. Brenner, R. Brenner, S. Bressler, D. L. Briglin, T. M. Bristow, D. Britton, D. Britzger, F. M. Brochu, I. Brock, R. Brock, G. Brooijmans, T. Brooks, W. K. Brooks, J. Brosamer, E. Brost, J. H Broughton, P. A. Bruckman de Renstrom, D. Bruncko, A. Bruni, G. Bruni, L. S. Bruni, S. Bruno, BH Brunt, M. Bruschi, N. Bruscino, P. Bryant, L. Bryngemark, T. Buanes, Q. Buat, P. Buchholz, A. G. Buckley, I. A. Budagov, F. Buehrer, M. K. Bugge, O. Bulekov, D. Bullock, T. J. Burch, S. Burdin, C. D. Burgard, A. M. Burger, B. Burghgrave, K. Burka, S. Burke, I. Burmeister, J. T. P. Burr, D. Büscher, V. Büscher, P. Bussey, J. M. Butler, C. M. Buttar, J. M. Butterworth, P. Butti, W. Buttinger, A. Buzatu, A. R. Buzykaev, C.-Q. Li, S. Cabrera Urbán, D. Caforio, H. Cai, V. M. Cairo, O. Cakir, N. Calace, P. Calafiura, A. Calandri, G. Calderini, P. Calfayan, G. Callea, L. P. Caloba, S. CalventeLopez, D. Calvet, S. Calvet, T. P. Calvet, R. Camacho Toro, S. Camarda, P. Camarri, D. Cameron, R. Caminal Armadans, C. Camincher, S. Campana, M. Campanelli, A. Camplani, A. Campoverde, V. Canale, M. Cano Bret, J. Cantero, T. Cao, M. D. M. Capeans Garrido, I. Caprini, M. Caprini, M. Capua, R. M. Carbone, R. Cardarelli, F. Cardillo, I. Carli, T. Carli, G. Carlino, B. T. Carlson, L. Carminati, R. M. D. Carney, S. Caron, E. Carquin, S. Carrá, G. D. Carrillo-Montoya, D. Casadei, M. P. Casado, A. F. Casha, M. Casolino, D. W. Casper, R. Castelijn, V. Castillo Gimenez, N. F. Castro, A. Catinaccio, J. R. Catmore, A. Cattai, J. Caudron, V. Cavaliere, E. Cavallaro, D. Cavalli, M. Cavalli-Sforza, V. Cavasinni, E. Celebi, F. Ceradini, L. Cerda Alberich, A. S. Cerqueira, A. Cerri, L. Cerrito, F. Cerutti, A. Cervelli, S. A. Cetin, A. Chafaq, D. Chakraborty, S. K. Chan, W. S. Chan, Y. L. Chan, P. Chang, J. D. Chapman, D. G. Charlton, C. C. Chau, C. A. Chavez Barajas, S. Che, S. Cheatham, A. Chegwidden, S. Chekanov, S. V. Chekulaev, G. A. Chelkov, M. A. Chelstowska, C. Chen, C. Chen, H. Chen, J. Chen, S. Chen, S. Chen, X. Chen, Y. Chen, H. C. Cheng, H. J. Cheng, A. Cheplakov, E. Cheremushkina, R. Cherkaoui El Moursli, E. Cheu, K. Cheung, L. Chevalier, V. Chiarella, G. Chiarelli, G. Chiodini, A. S. Chisholm, A. Chitan, Y. H. Chiu, M. V. Chizhov, K. Choi, A. R. Chomont, S. Chouridou, Y. S. Chow, V. Christodoulou, M. C. Chu, J. Chudoba, A. J. Chuinard, J. J. Chwastowski, L. Chytka, A. K. Ciftci, D. Cinca, V. Cindro, I. A. Cioara, A. Ciocio, F. Cirotto, Z. H. Citron, M. Citterio, M. Ciubancan, A. Clark, B. L. Clark, M. R. Clark, P. J. Clark, R. N. Clarke, C. Clement, Y. Coadou, M. Cobal, A. Coccaro, J. Cochran, L. Colasurdo, B. Cole, A. P. Colijn, J. Collot, T. Colombo, P. Conde Muiño, E. Coniavitis, S. H. Connell, I. A. Connelly, S. Constantinescu, G. Conti, F. Conventi, M. Cooke, A. M. Cooper-Sarkar, F. Cormier, K. J. R. Cormier, M. Corradi, F. Corriveau, A. Cortes-Gonzalez, G. Costa, M. J. Costa, D. Costanzo, G. Cottin, G. Cowan, B. E. Cox, K. Cranmer, S. J. Crawley, R. A. Creager, G. Cree, S. Crépé-Renaudin, F. Crescioli, W. A. Cribbs, M. Cristinziani, V. Croft, G. Crosetti, A. Cueto, T. Cuhadar Donszelmann, A. R. Cukierman, J. Cummings, M. Curatolo, J. Cúth, S. Czekierda, P. Czodrowski, G. D’amen, S. D’Auria, L. D’eramo, M. D’Onofrio, M. J. Da Cunha Sargedas De Sousa, C. DaVia, W. Dabrowski, T. Dado, T. Dai, O. Dale, F. Dallaire, C. Dallapiccola, M. Dam, J. R. Dandoy, M. F. Daneri, N. P. Dang, A. C. Daniells, N. S. Dann, M. Danninger, M. Dano Hoffmann, V. Dao, G. Darbo, S. Darmora, J. Dassoulas, A. Dattagupta, T. Daubney, W. Davey, C. David, T. Davidek, D. R. Davis, P. Davison, E. Dawe, I. Dawson, K. De, R. de Asmundis, A. De Benedetti, S. De Castro, S. De Cecco, N. De Groot, P. de Jong, H. De la Torre, F. De Lorenzi, A. De Maria, D. De Pedis, A. De Salvo, U. De Sanctis, A. De Santo, K. De Vasconcelos Corga, J. B. De Vivie De Regie, R. Debbe, C. Debenedetti, D. V. Dedovich, N. Dehghanian, I. Deigaard, M. Del Gaudio, J. Del Peso, D. Delgove, F. Deliot, C. M. Delitzsch, A. Dell’Acqua, L. Dell’Asta, M. Dell’Orso, M. Della Pietra, D. della Volpe, M. Delmastro, C. Delporte, P. A. Delsart, D. A. DeMarco, S. Demers, M. Demichev, A. Demilly, S. P. Denisov, D. Denysiuk, D. Derendarz, J. E. Derkaoui, F. Derue, P. Dervan, K. Desch, C. Deterre, K. Dette, M. R. Devesa, P. O. Deviveiros, A. Dewhurst, S. Dhaliwal, F. A. DiBello, A. DiCiaccio, L. DiCiaccio, W. K. DiClemente, C. DiDonato, A. DiGirolamo, B. DiGirolamo, B. DiMicco, R. DiNardo, K. F. DiPetrillo, A. Di Simone, R. Di Sipio, D. DiValentino, C. Diaconu, M. Diamond, F. A. Dias, M. A. Diaz, J. Dickinson, E. B. Diehl, J. Dietrich, S. Díez Cornell, A. Dimitrievska, J. Dingfelder, P. Dita, S. Dita, F. Dittus, F. Djama, T. Djobava, J. I. Djuvsland, M. A. B. doVale, D. Dobos, M. Dobre, D. Dodsworth, C. Doglioni, J. Dolejsi, Z. Dolezal, M. Donadelli, S. Donati, P. Dondero, J. Donini, J. Dopke, A. Doria, M. T. Dova, A. T. Doyle, E. Drechsler, M. Dris, Y. Du, J. Duarte-Campderros, F. Dubinin, A. Dubreuil, E. Duchovni, G. Duckeck, A. Ducourthial, O. A. Ducu, D. Duda, A. Dudarev, A. Chr. Dudder, E. M. Duffield, L. Duflot, M. Dührssen, C. Dulsen, M. Dumancic, A. E. Dumitriu, A. K. Duncan, M. Dunford, A. Duperrin, H. Duran Yildiz, M. Düren, A. Durglishvili, D. Duschinger, B. Dutta, D. Duvnjak, M. Dyndal, B. S. Dziedzic, C. Eckardt, K. M. Ecker, R. C. Edgar, T. Eifert, G. Eigen, K. Einsweiler, T. Ekelof, M. El Kacimi, R. El Kosseifi, V. Ellajosyula, M. Ellert, S. Elles, F. Ellinghaus, A. A. Elliot, N. Ellis, J. Elmsheuser, M. Elsing, D. Emeliyanov, Y. Enari, J. S. Ennis, M. B. Epland, J. Erdmann, A. Ereditato, M. Ernst, S. Errede, M. Escalier, C. Escobar, B. Esposito, O. EstradaPastor, A. I. Etienvre, E. Etzion, H. Evans, A. Ezhilov, M. Ezzi, F. Fabbri, L. Fabbri, V. Fabiani, G. Facini, R. M. Fakhrutdinov, S. Falciano, R. J. Falla, J. Faltova, Y. Fang, M. Fanti, A. Farbin, A. Farilla, C. Farina, E. M. Farina, T. Farooque, S. Farrell, S. M. Farrington, P. Farthouat, F. Fassi, P. Fassnacht, D. Fassouliotis, M. Faucci Giannelli, A. Favareto, W. J. Fawcett, L. Fayard, O. L. Fedin, W. Fedorko, S. Feigl, L. Feligioni, C. Feng, E. J. Feng, M. J. Fenton, A. B. Fenyuk, L. Feremenga, P. Fernandez Martinez, J. Ferrando, A. Ferrari, P. Ferrari, R. Ferrari, D. E. Ferreira de Lima, A. Ferrer, D. Ferrere, C. Ferretti, F. Fiedler, A. Filipčič, M. Filipuzzi, F. Filthaut, M. Fincke-Keeler, K. D. Finelli, M. C. N. Fiolhais, L. Fiorini, A. Fischer, C. Fischer, J. Fischer, W. C. Fisher, N. Flaschel, I. Fleck, P. Fleischmann, R. R. M. Fletcher, T. Flick, B. M. Flierl, L. R. FloresCastillo, M. J. Flowerdew, G. T. Forcolin, A. Formica, F. A. Förster, A. Forti, A. G. Foster, D. Fournier, H. Fox, S. Fracchia, P. Francavilla, M. Franchini, S. Franchino, D. Francis, L. Franconi, M. Franklin, M. Frate, M. Fraternali, D. Freeborn, S. M. Fressard-Batraneanu, B. Freund, D. Froidevaux, J. A. Frost, C. Fukunaga, T. Fusayasu, J. Fuster, O. Gabizon, A. Gabrielli, A. Gabrielli, G. P. Gach, S. Gadatsch, S. Gadomski, G. Gagliardi, L. G. Gagnon, C. Galea, B. Galhardo, E. J. Gallas, B. J. Gallop, P. Gallus, G. Galster, K. K. Gan, S. Ganguly, Y. Gao, Y. S. Gao, F. M. Garay Walls, C. García, J. E. García Navarro, J. A. García Pascual, M. Garcia-Sciveres, R. W. Gardner, N. Garelli, V. Garonne, A. GasconBravo, K. Gasnikova, C. Gatti, A. Gaudiello, G. Gaudio, I. L. Gavrilenko, C. Gay, G. Gaycken, E. N. Gazis, C. N. P. Gee, J. Geisen, M. Geisen, M. P. Geisler, K. Gellerstedt, C. Gemme, M. H. Genest, C. Geng, S. Gentile, C. Gentsos, S. George, D. Gerbaudo, G. Geßner, S. Ghasemi, M. Ghneimat, B. Giacobbe, S. Giagu, N. Giangiacomi, P. Giannetti, S. M. Gibson, M. Gignac, M. Gilchriese, D. Gillberg, G. Gilles, D. M. Gingrich, M. P. Giordani, F. M. Giorgi, P. F. Giraud, P. Giromini, G. Giugliarelli, D. Giugni, F. Giuli, C. Giuliani, M. Giulini, B. K. Gjelsten, S. Gkaitatzis, I. Gkialas, E. L. Gkougkousis, P. Gkountoumis, L. K. Gladilin, C. Glasman, J. Glatzer, P. C. F. Glaysher, A. Glazov, M. Goblirsch-Kolb, J. Godlewski, S. Goldfarb, T. Golling, D. Golubkov, A. Gomes, R. Gonçalo, R. Goncalves Gama, J. Goncalves Pinto Firmino Da Costa, G. Gonella, L. Gonella, A. Gongadze, J. L. Gonski, S. González de laHoz, S. Gonzalez-Sevilla, L. Goossens, P. A. Gorbounov, H. A. Gordon, I. Gorelov, B. Gorini, E. Gorini, A. Gorišek, A. T. Goshaw, C. Gössling, M. I. Gostkin, C. A. Gottardo, C. R. Goudet, D. Goujdami, A. G. Goussiou, N. Govender, E. Gozani, I. Grabowska-Bold, P. O. J. Gradin, J. Gramling, E. Gramstad, S. Grancagnolo, V. Gratchev, P. M. Gravila, C. Gray, H. M. Gray, Z. D. Greenwood, C. Grefe, K. Gregersen, I. M. Gregor, P. Grenier, K. Grevtsov, J. Griffiths, A. A. Grillo, K. Grimm, S. Grinstein, Ph. Gris, J.-F. Grivaz, S. Groh, E. Gross, J. Grosse-Knetter, G. C. Grossi, Z. J. Grout, A. Grummer, L. Guan, W. Guan, J. Guenther, F. Guescini, D. Guest, O. Gueta, B. Gui, E. Guido, T. Guillemin, S. Guindon, U. Gul, C. Gumpert, J. Guo, W. Guo, Y. Guo, R. Gupta, S. Gurbuz, G. Gustavino, B. J. Gutelman, P. Gutierrez, N. G. Gutierrez Ortiz, C. Gutschow, C. Guyot, M. P. Guzik, C. Gwenlan, C. B. Gwilliam, A. Haas, C. Haber, H. K. Hadavand, N. Haddad, A. Hadef, S. Hageböck, M. Hagihara, H. Hakobyan, M. Haleem, J. Haley, G. Halladjian, G. D. Hallewell, K. Hamacher, P. Hamal, K. Hamano, A. Hamilton, G. N. Hamity, P. G. Hamnett, L. Han, S. Han, K. Hanagaki, K. Hanawa, M. Hance, D. M. Handl, B. Haney, P. Hanke, J. B. Hansen, J. D. Hansen, M. C. Hansen, P. H. Hansen, K. Hara, A. S. Hard, T. Harenberg, F. Hariri, S. Harkusha, P. F. Harrison, N. M. Hartmann, Y. Hasegawa, A. Hasib, S. Hassani, S. Haug, R. Hauser, L. Hauswald, L. B. Havener, M. Havranek, C. M. Hawkes, R. J. Hawkings, D. Hayakawa, D. Hayden, C. P. Hays, J. M. Hays, H. S. Hayward, S. J. Haywood, S. J. Head, T. Heck, V. Hedberg, L. Heelan, S. Heer, K. K. Heidegger, S. Heim, T. Heim, B. Heinemann, J. J. Heinrich, L. Heinrich, C. Heinz, J. Hejbal, L. Helary, A. Held, S. Hellman, C. Helsens, R. C. W. Henderson, Y. Heng, S. Henkelmann, A. M. Henriques Correia, S. Henrot-Versille, G. H. Herbert, H. Herde, V. Herget, Y. Hernández Jiménez, H. Herr, G. Herten, R. Hertenberger, L. Hervas, T. C. Herwig, G. G. Hesketh, N. P. Hessey, J. W. Hetherly, S. Higashino, E. Higón-Rodriguez, K. Hildebrand, E. Hill, J. C. Hill, K. H. Hiller, S. J. Hillier, M. Hils, I. Hinchliffe, M. Hirose, D. Hirschbuehl, B. Hiti, O. Hladik, D. R. Hlaluku, X. Hoad, J. Hobbs, N. Hod, M. C. Hodgkinson, P. Hodgson, A. Hoecker, M. R. Hoeferkamp, F. Hoenig, D. Hohn, T. R. Holmes, M. Holzbock, M. Homann, S. Honda, T. Honda, T. M. Hong, B. H. Hooberman, W. H. Hopkins, Y. Horii, A. J. Horton, J-Y. Hostachy, A. Hostiuc, S. Hou, A. Hoummada, J. Howarth, J. Hoya, M. Hrabovsky, J. Hrdinka, I. Hristova, J. Hrivnac, T. Hryn’ova, A. Hrynevich, P. J. Hsu, S.-C. Hsu, Q. Hu, S. Hu, Y. Huang, Z. Hubacek, F. Hubaut, F. Huegging, T. B. Huffman, E. W. Hughes, M. Huhtinen, R. F. H. Hunter, P. Huo, N. Huseynov, J. Huston, J. Huth, R. Hyneman, G. Iacobucci, G. Iakovidis, I. Ibragimov, L. Iconomidou-Fayard, Z. Idrissi, P. Iengo, O. Igonkina, T. Iizawa, Y. Ikegami, M. Ikeno, Y. Ilchenko, D. Iliadis, N. Ilic, F. Iltzsche, G. Introzzi, P. Ioannou, M. Iodice, K. Iordanidou, V. Ippolito, M. F. Isacson, N. Ishijima, M. Ishino, M. Ishitsuka, C. Issever, S. Istin, F. Ito, J. M. Iturbe Ponce, R. Iuppa, H. Iwasaki, J. M. Izen, V. Izzo, S. Jabbar, P. Jackson, R. M. Jacobs, V. Jain, K. B. Jakobi, K. Jakobs, S. Jakobsen, T. Jakoubek, D. O. Jamin, D. K. Jana, R. Jansky, J. Janssen, M. Janus, P. A. Janus, G. Jarlskog, N. Javadov, T. Javůrek, M. Javurkova, F. Jeanneau, L. Jeanty, J. Jejelava, A. Jelinskas, P. Jenni, C. Jeske, S. Jézéquel, H. Ji, J. Jia, H. Jiang, Y. Jiang, Z. Jiang, S. Jiggins, J. Jimenez Pena, S. Jin, A. Jinaru, O. Jinnouchi, H. Jivan, P. Johansson, K. A. Johns, C. A. Johnson, W. J. Johnson, K. Jon-And, R. W. L. Jones, S. D. Jones, S. Jones, T. J. Jones, J. Jongmanns, P. M. Jorge, J. Jovicevic, X. Ju, A. JusteRozas, M. K. Köhler, A. Kaczmarska, M. Kado, H. Kagan, M. Kagan, S. J. Kahn, T. Kaji, E. Kajomovitz, C. W. Kalderon, A. Kaluza, S. Kama, A. Kamenshchikov, N. Kanaya, L. Kanjir, V. A. Kantserov, J. Kanzaki, B. Kaplan, L. S. Kaplan, D. Kar, K. Karakostas, N. Karastathis, M. J. Kareem, E. Karentzos, S. N. Karpov, Z. M. Karpova, K. Karthik, V. Kartvelishvili, A. N. Karyukhin, K. Kasahara, L. Kashif, R. D. Kass, A. Kastanas, Y. Kataoka, C. Kato, A. Katre, J. Katzy, K. Kawade, K. Kawagoe, T. Kawamoto, G. Kawamura, E. F. Kay, V. F. Kazanin, R. Keeler, R. Kehoe, J. S. Keller, E. Kellermann, J. J. Kempster, J Kendrick, H. Keoshkerian, O. Kepka, B. P. Kerševan, S. Kersten, R. A. Keyes, M. Khader, F. Khalil-zada, A. Khanov, A. G. Kharlamov, T. Kharlamova, A. Khodinov, T. J. Khoo, V. Khovanskiy, E. Khramov, J. Khubua, S. Kido, C. R. Kilby, H. Y. Kim, S. H. Kim, Y. K. Kim, N. Kimura, O. M. Kind, B. T. King, D. Kirchmeier, J. Kirk, A. E. Kiryunin, T. Kishimoto, D. Kisielewska, V. Kitali, O. Kivernyk, E. Kladiva, T. Klapdor-Kleingrothaus, M. H. Klein, M. Klein, U. Klein, K. Kleinknecht, P. Klimek, A. Klimentov, R. Klingenberg, T. Klingl, T. Klioutchnikova, F. F. Klitzner, E.-E. Kluge, P. Kluit, S. Kluth, E. Kneringer, E. B. F. G. Knoops, A. Knue, A. Kobayashi, D. Kobayashi, T. Kobayashi, M. Kobel, M. Kocian, P. Kodys, T. Koffas, E. Koffeman, N. M. Köhler, T. Koi, M. Kolb, I. Koletsou, T. Kondo, N. Kondrashova, K. Köneke, A. C. König, T. Kono, R. Konoplich, N. Konstantinidis, B. Konya, R. Kopeliansky, S. Koperny, A. K. Kopp, K. Korcyl, K. Kordas, A. Korn, A. A. Korol, I. Korolkov, E. V. Korolkova, O. Kortner, S. Kortner, T. Kosek, V. V. Kostyukhin, A. Kotwal, A. Koulouris, A. Kourkoumeli-Charalampidi, C. Kourkoumelis, E. Kourlitis, V. Kouskoura, A. B. Kowalewska, R. Kowalewski, T. Z. Kowalski, C. Kozakai, W. Kozanecki, A. S. Kozhin, V. A. Kramarenko, G. Kramberger, D. Krasnopevtsev, M. W. Krasny, A. Krasznahorkay, D. Krauss, J. A. Kremer, J. Kretzschmar, K. Kreutzfeldt, P. Krieger, K. Krizka, K. Kroeninger, H. Kroha, J. Kroll, J. Kroll, J. Kroseberg, J. Krstic, U. Kruchonak, H. Krüger, N. Krumnack, M. C. Kruse, T. Kubota, H. Kucuk, S. Kuday, J. T. Kuechler, S. Kuehn, A. Kugel, F. Kuger, T. Kuhl, V. Kukhtin, R. Kukla, Y. Kulchitsky, S. Kuleshov, Y. P. Kulinich, M. Kuna, T. Kunigo, A. Kupco, T. Kupfer, O. Kuprash, H. Kurashige, L. L. Kurchaninov, Y. A. Kurochkin, M. G. Kurth, E. S. Kuwertz, M. Kuze, J. Kvita, T. Kwan, D. Kyriazopoulos, A. LaRosa, J. L. La RosaNavarro, L. LaRotonda, F. LaRuffa, C. Lacasta, F. Lacava, J. Lacey, D. P. J. Lack, H. Lacker, D. Lacour, E. Ladygin, R. Lafaye, B. Laforge, T. Lagouri, S. Lai, S. Lammers, W. Lampl, E. Lançon, U. Landgraf, M. P. J. Landon, M. C. Lanfermann, V. S. Lang, J. C. Lange, R. J. Langenberg, A. J. Lankford, F. Lanni, K. Lantzsch, A. Lanza, A. Lapertosa, S. Laplace, J. F. Laporte, T. Lari, F. Lasagni Manghi, M. Lassnig, T. S. Lau, P. Laurelli, W. Lavrijsen, A. T. Law, P. Laycock, T. Lazovich, M. Lazzaroni, B. Le, O. Le Dortz, E. Le Guirriec, E. P. LeQuilleuc, M. LeBlanc, T. LeCompte, F. Ledroit-Guillon, C. A. Lee, G. R. Lee, S. C. Lee, L. Lee, B. Lefebvre, G. Lefebvre, M. Lefebvre, F. Legger, C. Leggett, G. Lehmann Miotto, X. Lei, W. A. Leight, M. A. L. Leite, R. Leitner, D. Lellouch, B. Lemmer, K. J. C. Leney, T. Lenz, B. Lenzi, R. Leone, S. Leone, C. Leonidopoulos, G. Lerner, C. Leroy, R. Les, A. A. J. Lesage, C. G. Lester, M. Levchenko, J. Levêque, D. Levin, L. J. Levinson, M. Levy, D. Lewis, B. Li, C.-Q. Li, H. Li, L. Li, Q. Li, Q. Li, S. Li, X. Li, Y. Li, Z. Liang, B. Liberti, A. Liblong, K. Lie, J. Liebal, W. Liebig, A. Limosani, C. Y. Lin, K. Lin, S. C. Lin, T. H. Lin, R. A. Linck, B. E. Lindquist, A. E. Lionti, E. Lipeles, A. Lipniacka, M. Lisovyi, T. M. Liss, A. Lister, A. M. Litke, B. Liu, H. Liu, H. Liu, J. K. K. Liu, J. Liu, J. B. Liu, K. Liu, L. Liu, M. Liu, Y. L. Liu, Y. Liu, M. Livan, A. Lleres, J. LlorenteMerino, S. L. Lloyd, C. Y. Lo, F. Lo Sterzo, E. M. Lobodzinska, P. Loch, F. K. Loebinger, A. Loesle, K. M. Loew, T. Lohse, K. Lohwasser, M. Lokajicek, B. A. Long, J. D. Long, R. E. Long, L. Longo, K. A. Looper, J. A. Lopez, I. Lopez Paz, A. Lopez Solis, J. Lorenz, N. Lorenzo Martinez, M. Losada, P. J. Lösel, X. Lou, A. Lounis, J. Love, P. A. Love, H. Lu, N. Lu, Y. J. Lu, H. J. Lubatti, C. Luci, A. Lucotte, C. Luedtke, F. Luehring, W. Lukas, L. Luminari, O. Lundberg, B. Lund-Jensen, M. S. Lutz, P. M. Luzi, D. Lynn, R. Lysak, E. Lytken, F. Lyu, V. Lyubushkin, H. Ma, L. L. Ma, Y. Ma, G. Maccarrone, A. Macchiolo, C. M. Macdonald, B. Maček, J. Machado Miguens, D. Madaffari, R. Madar, W. F. Mader, A. Madsen, N. Madysa, J. Maeda, S. Maeland, T. Maeno, A. S. Maevskiy, V. Magerl, C. Maiani, C. Maidantchik, T. Maier, A. Maio, O. Majersky, S. Majewski, Y. Makida, N. Makovec, B. Malaescu, Pa. Malecki, V. P. Maleev, F. Malek, U. Mallik, D. Malon, C. Malone, S. Maltezos, S. Malyukov, J. Mamuzic, G. Mancini, I. Mandić, J. Maneira, L. Manhaes de Andrade Filho, J. Manjarres Ramos, K. H. Mankinen, A. Mann, A. Manousos, B. Mansoulie, J. D. Mansour, R. Mantifel, M. Mantoani, S. Manzoni, L. Mapelli, G. Marceca, L. March, L. Marchese, G. Marchiori, M. Marcisovsky, C. A. Marin Tobon, M. Marjanovic, D. E. Marley, F. Marroquim, S. P. Marsden, Z. Marshall, M. U. F Martensson, S. Marti-Garcia, C. B. Martin, T. A. Martin, V. J. Martin, B. Martin dit Latour, M. Martinez, V. I. Martinez Outschoorn, S. Martin-Haugh, V. S. Martoiu, A. C. Martyniuk, A. Marzin, L. Masetti, T. Mashimo, R. Mashinistov, J. Masik, A. L. Maslennikov, L. H. Mason, L. Massa, P. Mastrandrea, A. Mastroberardino, T. Masubuchi, P. Mättig, J. Maurer, S. J. Maxfield, D. A. Maximov, R. Mazini, I. Maznas, S. M. Mazza, N. C. Mc Fadden, G. Mc Goldrick, S. P. Mc Kee, A. McCarn, R. L. McCarthy, T. G. McCarthy, L. I. McClymont, E. F. McDonald, J. A. Mcfayden, G. Mchedlidze, S. J. McMahon, P. C. McNamara, C. J. McNicol, R. A. McPherson, S. Meehan, T. J. Megy, S. Mehlhase, A. Mehta, T. Meideck, K. Meier, B. Meirose, D. Melini, B. R. Mellado Garcia, J. D. Mellenthin, M. Melo, F. Meloni, A. Melzer, S. B. Menary, L. Meng, X. T. Meng, A. Mengarelli, S. Menke, E. Meoni, S. Mergelmeyer, C. Merlassino, P. Mermod, L. Merola, C. Meroni, F. S. Merritt, A. Messina, J. Metcalfe, A. S. Mete, C. Meyer, J-P. Meyer, J. Meyer, H. Meyer Zu Theenhausen, F. Miano, R. P. Middleton, S. Miglioranzi, L. Mijović, G. Mikenberg, M. Mikestikova, M. Mikuž, M. Milesi, A. Milic, D. A. Millar, D. W. Miller, C. Mills, A. Milov, D. A. Milstead, A. A. Minaenko, Y. Minami, I. A. Minashvili, A. I. Mincer, B. Mindur, M. Mineev, Y. Minegishi, Y. Ming, L. M. Mir, A. Mirto, K. P. Mistry, T. Mitani, J. Mitrevski, V. A. Mitsou, A. Miucci, P. S. Miyagawa, A. Mizukami, J. U. Mjörnmark, T. Mkrtchyan, M. Mlynarikova, T. Moa, K. Mochizuki, P. Mogg, S. Mohapatra, S. Molander, R. Moles-Valls, M. C. Mondragon, K. Mönig, J. Monk, E. Monnier, A. Montalbano, J. Montejo Berlingen, F. Monticelli, S. Monzani, R. W. Moore, N. Morange, D. Moreno, M. Moreno Llácer, P. Morettini, S. Morgenstern, D. Mori, T. Mori, M. Morii, M. Morinaga, V. Morisbak, A. K. Morley, G. Mornacchi, J. D. Morris, L. Morvaj, P. Moschovakos, M. Mosidze, H. J. Moss, J. Moss, K. Motohashi, R. Mount, E. Mountricha, E. J. W. Moyse, S. Muanza, F. Mueller, J. Mueller, R. S. P. Mueller, D. Muenstermann, P. Mullen, G. A. Mullier, F. J. MunozSanchez, W. J. Murray, H. Musheghyan, M. Muškinja, A. G. Myagkov, M. Myska, B. P. Nachman, O. Nackenhorst, K. Nagai, R. Nagai, K. Nagano, Y. Nagasaka, K. Nagata, M. Nagel, E. Nagy, A. M. Nairz, Y. Nakahama, K. Nakamura, T. Nakamura, I. Nakano, R. F. NaranjoGarcia, R. Narayan, D. I. Narrias Villar, I. Naryshkin, T. Naumann, G. Navarro, R. Nayyar, H. A. Neal, P. Yu. Nechaeva, T. J. Neep, A. Negri, M. Negrini, S. Nektarijevic, C. Nellist, A. Nelson, M. E. Nelson, S. Nemecek, P. Nemethy, M. Nessi, M. S. Neubauer, M. Neumann, P. R. Newman, T. Y. Ng, Y. S. Ng, T. Nguyen Manh, R. B. Nickerson, R. Nicolaidou, J. Nielsen, N. Nikiforou, V. Nikolaenko, I. Nikolic-Audit, K. Nikolopoulos, P. Nilsson, Y. Ninomiya, A. Nisati, N. Nishu, R. Nisius, I. Nitsche, T. Nitta, T. Nobe, Y. Noguchi, M. Nomachi, I. Nomidis, M. A. Nomura, T. Nooney, M. Nordberg, N. Norjoharuddeen, O. Novgorodova, M. Nozaki, L. Nozka, K. Ntekas, E. Nurse, F. Nuti, K. O’connor, D. C. O’Neil, A. A. O’Rourke, V. O’Shea, F. G. Oakham, H. Oberlack, T. Obermann, J. Ocariz, A. Ochi, I. Ochoa, J. P. Ochoa-Ricoux, S. Oda, S. Odaka, A. Oh, S. H. Oh, C. C. Ohm, H. Ohman, H. Oide, H. Okawa, Y. Okumura, T. Okuyama, A. Olariu, L. F. OleiroSeabra, S. A. OlivaresPino, D. Oliveira Damazio, M. J. R. Olsson, A. Olszewski, J. Olszowska, A. Onofre, K. Onogi, P. U. E. Onyisi, H. Oppen, M. J. Oreglia, Y. Oren, D. Orestano, N. Orlando, R. S. Orr, B. Osculati, R. Ospanov, G. Otero y Garzon, H. Otono, M. Ouchrif, F. Ould-Saada, A. Ouraou, K. P. Oussoren, Q. Ouyang, M. Owen, R. E. Owen, V. E. Ozcan, N. Ozturk, K. Pachal, A. Pacheco Pages, L. Pacheco Rodriguez, C. Padilla Aranda, S. Pagan Griso, M. Paganini, F. Paige, G. Palacino, S. Palazzo, S. Palestini, M. Palka, D. Pallin, E. St. Panagiotopoulou, I. Panagoulias, C. E. Pandini, J. G. PanduroVazquez, P. Pani, S. Panitkin, D. Pantea, L. Paolozzi, Th. D. Papadopoulou, K. Papageorgiou, A. Paramonov, D. ParedesHernandez, A. J. Parker, M. A. Parker, K. A. Parker, F. Parodi, J. A. Parsons, U. Parzefall, V. R. Pascuzzi, J. M. Pasner, E. Pasqualucci, S. Passaggio, Fr. Pastore, S. Pataraia, J. R. Pater, T. Pauly, B. Pearson, S. Pedraza Lopez, R. Pedro, S. V. Peleganchuk, O. Penc, C. Peng, H. Peng, J. Penwell, B. S. Peralva, M. M. Perego, D. V. Perepelitsa, F. Peri, L. Perini, H. Pernegger, S. Perrella, R. Peschke, V. D. Peshekhonov, K. Peters, R. F. Y. Peters, B. A. Petersen, T. C. Petersen, E. Petit, A. Petridis, C. Petridou, P. Petroff, E. Petrolo, M. Petrov, F. Petrucci, N. E. Pettersson, A. Peyaud, R. Pezoa, F. H. Phillips, P. W. Phillips, G. Piacquadio, E. Pianori, A. Picazio, M. A. Pickering, R. Piegaia, J. E. Pilcher, A. D. Pilkington, M. Pinamonti, J. L. Pinfold, H. Pirumov, M. Pitt, L. Plazak, M.-A. Pleier, V. Pleskot, E. Plotnikova, D. Pluth, P. Podberezko, R. Poettgen, R. Poggi, L. Poggioli, I. Pogrebnyak, D. Pohl, I. Pokharel, G. Polesello, A. Poley, A. Policicchio, R. Polifka, A. Polini, C. S. Pollard, V. Polychronakos, K. Pommès, D. Ponomarenko, L. Pontecorvo, G. A. Popeneciu, D. M. Portillo Quintero, S. Pospisil, K. Potamianos, I. N. Potrap, C. J. Potter, H. Potti, T. Poulsen, J. Poveda, M. E. Pozo Astigarraga, P. Pralavorio, A. Pranko, S. Prell, D. Price, M. Primavera, S. Prince, N. Proklova, K. Prokofiev, F. Prokoshin, S. Protopopescu, J. Proudfoot, M. Przybycien, A. Puri, P. Puzo, J. Qian, G. Qin, Y. Qin, A. Quadt, M. Queitsch-Maitland, D. Quilty, S. Raddum, V. Radeka, V. Radescu, S. K. Radhakrishnan, P. Radloff, P. Rados, F. Ragusa, G. Rahal, J. A. Raine, S. Rajagopalan, C. Rangel-Smith, T. Rashid, S. Raspopov, M. G. Ratti, D. M. Rauch, F. Rauscher, S. Rave, I. Ravinovich, J. H. Rawling, M. Raymond, A. L. Read, N. P. Readioff, M. Reale, D. M. Rebuzzi, A. Redelbach, G. Redlinger, R. Reece, R. G. Reed, K. Reeves, L. Rehnisch, J. Reichert, A. Reiss, C. Rembser, H. Ren, M. Rescigno, S. Resconi, E. D. Resseguie, S. Rettie, E. Reynolds, O. L. Rezanova, P. Reznicek, R. Rezvani, R. Richter, S. Richter, E. Richter-Was, O. Ricken, M. Ridel, P. Rieck, C. J. Riegel, J. Rieger, O. Rifki, M. Rijssenbeek, A. Rimoldi, M. Rimoldi, L. Rinaldi, G. Ripellino, B. Ristić, E. Ritsch, I. Riu, F. Rizatdinova, E. Rizvi, C. Rizzi, R. T. Roberts, S. H. Robertson, A. Robichaud-Veronneau, D. Robinson, J. E. M. Robinson, A. Robson, E. Rocco, C. Roda, Y. Rodina, S. RodriguezBosca, A. RodriguezPerez, D. RodriguezRodriguez, S. Roe, C. S. Rogan, O. Røhne, J. Roloff, A. Romaniouk, M. Romano, S. M. Romano Saez, E. Romero Adam, N. Rompotis, M. Ronzani, L. Roos, S. Rosati, K. Rosbach, P. Rose, N.-A. Rosien, E. Rossi, L. P. Rossi, J. H. N. Rosten, R. Rosten, M. Rotaru, J. Rothberg, D. Rousseau, D. Roy, A. Rozanov, Y. Rozen, X. Ruan, F. Rubbo, F. Rühr, A. Ruiz-Martinez, Z. Rurikova, N. A. Rusakovich, H. L. Russell, J. P. Rutherfoord, N. Ruthmann, E. M. Rüttinger, Y. F. Ryabov, M. Rybar, G. Rybkin, S. Ryu, A. Ryzhov, G. F. Rzehorz, A. F. Saavedra, G. Sabato, S. Sacerdoti, H.F-W. Sadrozinski, R. Sadykov, F. Safai Tehrani, P. Saha, M. Sahinsoy, M. Saimpert, M. Saito, T. Saito, H. Sakamoto, Y. Sakurai, G. Salamanna, J. E. Salazar Loyola, D. Salek, P. H. Sales De Bruin, D. Salihagic, A. Salnikov, J. Salt, D. Salvatore, F. Salvatore, A. Salvucci, A. Salzburger, D. Sammel, D. Sampsonidis, D. Sampsonidou, J. Sánchez, V. Sanchez Martinez, A. Sanchez Pineda, H. Sandaker, R. L. Sandbach, C. O. Sander, M. Sandhoff, C. Sandoval, D. P. C. Sankey, M. Sannino, Y. Sano, A. Sansoni, C. Santoni, H. Santos, I. Santoyo Castillo, A. Sapronov, J. G. Saraiva, B. Sarrazin, O. Sasaki, K. Sato, E. Sauvan, G. Savage, P. Savard, N. Savic, C. Sawyer, L. Sawyer, J. Saxon, C. Sbarra, A. Sbrizzi, T. Scanlon, D. A. Scannicchio, J. Schaarschmidt, P. Schacht, B. M. Schachtner, D. Schaefer, L. Schaefer, R. Schaefer, J. Schaeffer, S. Schaepe, S. Schaetzel, U. Schäfer, A. C. Schaffer, D. Schaile, R. D. Schamberger, V. A. Schegelsky, D. Scheirich, F. Schenck, M. Schernau, C. Schiavi, S. Schier, L. K. Schildgen, C. Schillo, M. Schioppa, S. Schlenker, K. R. Schmidt-Sommerfeld, K. Schmieden, C. Schmitt, S. Schmitt, S. Schmitz, U. Schnoor, L. Schoeffel, A. Schoening, B. D. Schoenrock, E. Schopf, M. Schott, J. F. P. Schouwenberg, J. Schovancova, S. Schramm, N. Schuh, A. Schulte, M. J. Schultens, H.-C. Schultz-Coulon, H. Schulz, M. Schumacher, B. A. Schumm, Ph. Schune, A. Schwartzman, T. A. Schwarz, H. Schweiger, Ph. Schwemling, R. Schwienhorst, J. Schwindling, A. Sciandra, G. Sciolla, M. Scornajenghi, F. Scuri, F. Scutti, J. Searcy, P. Seema, S. C. Seidel, A. Seiden, J. M. Seixas, G. Sekhniaidze, K. Sekhon, S. J. Sekula, N. Semprini-Cesari, S. Senkin, C. Serfon, L. Serin, L. Serkin, M. Sessa, R. Seuster, H. Severini, T. Šfiligoj, F. Sforza, A. Sfyrla, E. Shabalina, N. W. Shaikh, L. Y. Shan, R. Shang, J. T. Shank, M. Shapiro, P. B. Shatalov, K. Shaw, S. M. Shaw, A. Shcherbakova, C. Y. Shehu, Y. Shen, N. Sherafati, A. D. Sherman, P. Sherwood, L. Shi, S. Shimizu, C. O. Shimmin, M. Shimojima, I. P. J. Shipsey, S. Shirabe, M. Shiyakova, J. Shlomi, A. Shmeleva, D. Shoaleh Saadi, M. J. Shochet, S. Shojaii, D. R. Shope, S. Shrestha, E. Shulga, M. A. Shupe, P. Sicho, A. M. Sickles, P. E. Sidebo, E. Sideras Haddad, O. Sidiropoulou, A. Sidoti, F. Siegert, Dj. Sijacki, J. Silva, S. B. Silverstein, V. Simak, L. Simic, S. Simion, E. Simioni, B. Simmons, M. Simon, P. Sinervo, N. B. Sinev, M. Sioli, G. Siragusa, I. Siral, S. Yu. Sivoklokov, J. Sjölin, M. B. Skinner, P. Skubic, M. Slater, T. Slavicek, M. Slawinska, K. Sliwa, R. Slovak, V. Smakhtin, B. H. Smart, J. Smiesko, N. Smirnov, S. Yu. Smirnov, Y. Smirnov, L. N. Smirnova, O. Smirnova, J. W. Smith, M. N. K. Smith, R. W. Smith, M. Smizanska, K. Smolek, A. A. Snesarev, I. M. Snyder, S. Snyder, R. Sobie, F. Socher, A. Soffer, A. Søgaard, D. A. Soh, G. Sokhrannyi, C. A. SolansSanchez, M. Solar, E. Yu. Soldatov, U. Soldevila, A. A. Solodkov, A. Soloshenko, O. V. Solovyanov, V. Solovyev, P. Sommer, H. Son, A. Sopczak, D. Sosa, C. L. Sotiropoulou, S. Sottocornola, R. Soualah, A. M. Soukharev, D. South, B. C. Sowden, S. Spagnolo, M. Spalla, M. Spangenberg, F. Spanò, D. Sperlich, F. Spettel, T. M. Spieker, R. Spighi, G. Spigo, L. A. Spiller, M. Spousta, R. D. St.Denis, A. Stabile, R. Stamen, S. Stamm, E. Stanecka, R. W. Stanek, C. Stanescu, M. M. Stanitzki, B. S. Stapf, S. Stapnes, E. A. Starchenko, G. H. Stark, J. Stark, S. H Stark, P. Staroba, P. Starovoitov, S. Stärz, R. Staszewski, M. Stegler, P. Steinberg, B. Stelzer, H. J. Stelzer, O. Stelzer-Chilton, H. Stenzel, T. J. Stevenson, G. A. Stewart, M. C. Stockton, M. Stoebe, G. Stoicea, P. Stolte, S. Stonjek, A. R. Stradling, A. Straessner, M. E. Stramaglia, J. Strandberg, S. Strandberg, M. Strauss, P. Strizenec, R. Ströhmer, D. M. Strom, R. Stroynowski, A. Strubig, S. A. Stucci, B. Stugu, N. A. Styles, D. Su, J. Su, S. Suchek, Y. Sugaya, M. Suk, V. V. Sulin, DMS Sultan, S. Sultansoy, T. Sumida, S. Sun, X. Sun, K. Suruliz, C. J. E. Suster, M. R. Sutton, S. Suzuki, M. Svatos, M. Swiatlowski, S. P. Swift, I. Sykora, T. Sykora, D. Ta, K. Tackmann, J. Taenzer, A. Taffard, R. Tafirout, E. Tahirovic, N. Taiblum, H. Takai, R. Takashima, E. H. Takasugi, K. Takeda, T. Takeshita, Y. Takubo, M. Talby, A. A. Talyshev, J. Tanaka, M. Tanaka, R. Tanaka, S. Tanaka, R. Tanioka, B. B. Tannenwald, S. Tapia Araya, S. Tapprogge, S. Tarem, G. F. Tartarelli, P. Tas, M. Tasevsky, T. Tashiro, E. Tassi, A. Tavares Delgado, Y. Tayalati, A. C. Taylor, A. J. Taylor, G. N. Taylor, P. T. E. Taylor, W. Taylor, P. Teixeira-Dias, D. Temple, H. TenKate, P. K. Teng, J. J. Teoh, F. Tepel, S. Terada, K. Terashi, J. Terron, S. Terzo, M. Testa, R. J. Teuscher, S. J. Thais, T. Theveneaux-Pelzer, F. Thiele, J. P. Thomas, J. Thomas-Wilsker, P. D. Thompson, A. S. Thompson, L. A. Thomsen, E. Thomson, Y. Tian, M. J. Tibbetts, R. E. TicseTorres, V. O. Tikhomirov, Yu. A. Tikhonov, S. Timoshenko, P. Tipton, S. Tisserant, K. Todome, S. Todorova-Nova, S. Todt, J. Tojo, S. Tokár, K. Tokushuku, E. Tolley, L. Tomlinson, M. Tomoto, L. Tompkins, K. Toms, B. Tong, P. Tornambe, E. Torrence, H. Torres, E. TorróPastor, J. Toth, F. Touchard, D. R. Tovey, C. J. Treado, T. Trefzger, F. Tresoldi, A. Tricoli, I. M. Trigger, S. Trincaz-Duvoid, M. F. Tripiana, W. Trischuk, B. Trocmé, A. Trofymov, C. Troncon, M. Trottier-McDonald, M. Trovatelli, L. Truong, M. Trzebinski, A. Trzupek, K. W. Tsang, J.C-L. Tseng, P. V. Tsiareshka, N. Tsirintanis, S. Tsiskaridze, V. Tsiskaridze, E. G. Tskhadadze, I. I. Tsukerman, V. Tsulaia, S. Tsuno, D. Tsybychev, Y. Tu, A. Tudorache, V. Tudorache, T. T. Tulbure, A. N. Tuna, S. Turchikhin, D. Turgeman, I. TurkCakir, R. Turra, P. M. Tuts, G. Ucchielli, I. Ueda, M. Ughetto, F. Ukegawa, G. Unal, A. Undrus, G. Unel, F. C. Ungaro, Y. Unno, K. Uno, C. Unverdorben, J. Urban, P. Urquijo, P. Urrejola, G. Usai, J. Usui, L. Vacavant, V. Vacek, B. Vachon, K. O. H. Vadla, A. Vaidya, C. Valderanis, E. Valdes Santurio, M. Valente, S. Valentinetti, A. Valero, L. Valéry, S. Valkar, A. Vallier, J. A. Valls Ferrer, W. Van Den Wollenberg, H. van der Graaf, P. van Gemmeren, J. Van Nieuwkoop, I. van Vulpen, M. C. van Woerden, M. Vanadia, W. Vandelli, A. Vaniachine, P. Vankov, G. Vardanyan, R. Vari, E. W. Varnes, C. Varni, T. Varol, D. Varouchas, A. Vartapetian, K. E. Varvell, J. G. Vasquez, G. A. Vasquez, F. Vazeille, D. Vazquez Furelos, T. Vazquez Schroeder, J. Veatch, V. Veeraraghavan, L. M. Veloce, F. Veloso, S. Veneziano, A. Ventura, M. Venturi, N. Venturi, A. Venturini, V. Vercesi, M. Verducci, W. Verkerke, A. T. Vermeulen, J. C. Vermeulen, M. C. Vetterli, N. Viaux Maira, O. Viazlo, I. Vichou, T. Vickey, O. E. Vickey Boeriu, G. H. A. Viehhauser, S. Viel, L. Vigani, M. Villa, M. VillaplanaPerez, E. Vilucchi, M. G. Vincter, V. B. Vinogradov, A. Vishwakarma, C. Vittori, I. Vivarelli, S. Vlachos, M. Vogel, P. Vokac, G. Volpi, H. von der Schmitt, E. von Toerne, V. Vorobel, K. Vorobev, M. Vos, R. Voss, J. H. Vossebeld, N. Vranjes, M. Vranjes Milosavljevic, V. Vrba, M. Vreeswijk, R. Vuillermet, I. Vukotic, P. Wagner, W. Wagner, J. Wagner-Kuhr, H. Wahlberg, S. Wahrmund, K. Wakamiya, J. Walder, R. Walker, W. Walkowiak, V. Wallangen, C. Wang, C. Wang, F. Wang, H. Wang, H. Wang, J. Wang, J. Wang, Q. Wang, R.-J. Wang, R. Wang, S. M. Wang, T. Wang, W. Wang, W. Wang, Z. Wang, C. Wanotayaroj, A. Warburton, C. P. Ward, D. R. Wardrope, A. Washbrook, P. M. Watkins, A. T. Watson, M. F. Watson, G. Watts, S. Watts, B. M. Waugh, A. F. Webb, S. Webb, M. S. Weber, S. M. Weber, S. W. Weber, S. A. Weber, J. S. Webster, A. R. Weidberg, B. Weinert, J. Weingarten, M. Weirich, C. Weiser, H. Weits, P. S. Wells, T. Wenaus, T. Wengler, S. Wenig, N. Wermes, M. D. Werner, P. Werner, M. Wessels, T. D. Weston, K. Whalen, N. L. Whallon, A. M. Wharton, A. S. White, A. White, M. J. White, R. White, D. Whiteson, B. W. Whitmore, F. J. Wickens, W. Wiedenmann, M. Wielers, C. Wiglesworth, L. A. M. Wiik-Fuchs, A. Wildauer, F. Wilk, H. G. Wilkens, H. H. Williams, S. Williams, C. Willis, S. Willocq, J. A. Wilson, I. Wingerter-Seez, E. Winkels, F. Winklmeier, O. J. Winston, B. T. Winter, M. Wittgen, M. Wobisch, A. Wolf, T. M. H. Wolf, R. Wolff, M. W. Wolter, H. Wolters, V. W. S. Wong, N. L. Woods, S. D. Worm, B. K. Wosiek, J. Wotschack, K. W. Wozniak, M. Wu, S. L. Wu, X. Wu, Y. Wu, T. R. Wyatt, B. M. Wynne, S. Xella, Z. Xi, L. Xia, D. Xu, L. Xu, T. Xu, W. Xu, B. Yabsley, S. Yacoob, D. Yamaguchi, Y. Yamaguchi, A. Yamamoto, S. Yamamoto, T. Yamanaka, F. Yamane, M. Yamatani, T. Yamazaki, Y. Yamazaki, Z. Yan, H. Yang, H. Yang, Y. Yang, Z. Yang, W-M. Yao, Y. C. Yap, Y. Yasu, E. Yatsenko, K. H. YauWong, J. Ye, S. Ye, I. Yeletskikh, E. Yigitbasi, E. Yildirim, K. Yorita, K. Yoshihara, C. Young, C. J. S. Young, J. Yu, J. Yu, S. P. Y. Yuen, I. Yusuff, B. Zabinski, G. Zacharis, R. Zaidan, A. M. Zaitsev, N. Zakharchuk, J. Zalieckas, A. Zaman, S. Zambito, D. Zanzi, C. Zeitnitz, G. Zemaityte, A. Zemla, J. C. Zeng, Q. Zeng, O. Zenin, T. Ženiš, D. Zerwas, D. Zhang, D. Zhang, F. Zhang, G. Zhang, H. Zhang, J. Zhang, L. Zhang, L. Zhang, M. Zhang, P. Zhang, R. Zhang, R. Zhang, X. Zhang, Y. Zhang, Z. Zhang, X. Zhao, Y. Zhao, Z. Zhao, A. Zhemchugov, B. Zhou, C. Zhou, L. Zhou, M. Zhou, M. Zhou, N. Zhou, Y. Zhou, C. G. Zhu, H. Zhu, J. Zhu, Y. Zhu, X. Zhuang, K. Zhukov, A. Zibell, D. Zieminska, N. I. Zimine, C. Zimmermann, S. Zimmermann, Z. Zinonos, M. Zinser, M. Ziolkowski, L. Živković, G. Zobernig, A. Zoccoli, R. Zou, M. zur Nedden, L. Zwalinski, ATLAS Collaboration

**Affiliations:** 1 Department of Physics, University of Adelaide, Adelaide, Australia; 2 Physics Department, SUNY Albany, Albany, NY USA; 3 Department of Physics, University of Alberta, Edmonton, AB Canada; 4 Department of Physics, Ankara University, Ankara, Turkey; 5 Istanbul Aydin University, Istanbul, Turkey; 6 Division of Physics, TOBB University of Economics and Technology, Ankara, Turkey; 7 LAPP, CNRS/IN2P3 and Université Savoie Mont Blanc, Annecy-le-Vieux, France; 8 High Energy Physics Division, Argonne National Laboratory, Argonne, IL USA; 9 Department of Physics, University of Arizona, Tucson, AZ USA; 10 Department of Physics, The University of Texas at Arlington, Arlington, TX USA; 11 Physics Department, National and Kapodistrian University of Athens, Athens, Greece; 12 Physics Department, National Technical University of Athens, Zografou, Greece; 13 Department of Physics, The University of Texas at Austin, Austin, TX USA; 14 Institute of Physics, Azerbaijan Academy of Sciences, Baku, Azerbaijan; 15 Institut de Física d’Altes Energies (IFAE), The Barcelona Institute of Science and Technology, Barcelona, Spain; 16 Institute of Physics, University of Belgrade, Belgrade, Serbia; 17 Department for Physics and Technology, University of Bergen, Bergen, Norway; 18 Physics Division, Lawrence Berkeley National Laboratory, University of California, Berkeley, CA USA; 19 Department of Physics, Humboldt University, Berlin, Germany; 20 Albert Einstein Center for Fundamental Physics, Laboratory for High Energy Physics, University of Bern, Bern, Switzerland; 21 School of Physics and Astronomy, University of Birmingham, Birmingham, UK; 22 Department of Physics, Bogazici University, Istanbul, Turkey; 23 Department of Physics Engineering, Gaziantep University, Gaziantep, Turkey; 24 Faculty of Engineering and Natural Sciences, Istanbul Bilgi University, Istanbul, Turkey; 25 Faculty of Engineering and Natural Sciences, Bahcesehir University, Istanbul, Turkey; 26 Centro de Investigaciones, Universidad Antonio Narino, Bogotá, Colombia; 27 INFN Sezione di Bologna, Bologna, Italy; 28 Dipartimento di Fisica e Astronomia, Università di Bologna, Bologna, Italy; 29 Physikalisches Institut, University of Bonn, Bonn, Germany; 30 Department of Physics, Boston University, Boston, MA USA; 31 Department of Physics, Brandeis University, Waltham, MA USA; 32 Universidade Federal do Rio De Janeiro COPPE/EE/IF, Rio de Janeiro, Brazil; 33 Electrical Circuits Department, Federal University of Juiz de Fora (UFJF), Juiz de Fora, Brazil; 34 Federal University of Sao Joao del Rei (UFSJ), Sao Joao del Rei, Brazil; 35 Instituto de Fisica, Universidade de Sao Paulo, São Paulo, Brazil; 36 Physics Department, Brookhaven National Laboratory, Upton, NY USA; 37 Transilvania University of Brasov, Brasov, Romania; 38 Horia Hulubei National Institute of Physics and Nuclear Engineering, Bucharest, Romania; 39 Department of Physics, Alexandru Ioan Cuza University of Iasi, Iasi, Romania; 40 Physics Department, National Institute for Research and Development of Isotopic and Molecular Technologies, Cluj Napoca, Romania; 41 University Politehnica Bucharest, Bucharest, Romania; 42 West University in Timisoara, Timisoara, Romania; 43 Departamento de Física, Universidad de Buenos Aires, Buenos Aires, Argentina; 44 Cavendish Laboratory, University of Cambridge, Cambridge, UK; 45 Department of Physics, Carleton University, Ottawa, ON Canada; 46 CERN, Geneva, Switzerland; 47 Enrico Fermi Institute, University of Chicago, Chicago, IL USA; 48 Departamento de Física, Pontificia Universidad Católica de Chile, Santiago, Chile; 49 Departamento de Física, Universidad Técnica Federico Santa María, Valparaíso, Chile; 50 Institute of High Energy Physics, Chinese Academy of Sciences, Beijing, China; 51 Department of Physics, Nanjing University, Nanjing, Jiangsu China; 52 Physics Department, Tsinghua University, Beijing, 100084 China; 53 University of Chinese Academy of Science (UCAS), Beijing, China; 54 Department of Modern Physics and State Key Laboratory of Particle Detection and Electronics, University of Science and Technology of China, Anhui, China; 55 School of Physics, Shandong University, Shandong, China; 56 School of Physics and Astronomy, Key Laboratory for Particle Physics, Astrophysics and Cosmology, Ministry of Education; Shanghai Key Laboratory for Particle Physics and Cosmology, Tsung-Dao Lee Institute, Shanghai Jiao Tong University, Shanghai Shi, China; 57 Université Clermont Auvergne, CNRS/IN2P3, LPC, Clermont-Ferrand, France; 58 Nevis Laboratory, Columbia University, Irvington, NY USA; 59 Niels Bohr Institute, University of Copenhagen, Kobenhavn, Denmark; 60 INFN Gruppo Collegato di Cosenza, Laboratori Nazionali di Frascati, Frascati, Italy; 61 Dipartimento di Fisica, Università della Calabria, Rende, Italy; 62 Faculty of Physics and Applied Computer Science, AGH University of Science and Technology, Krakow, Poland; 63 Marian Smoluchowski Institute of Physics, Jagiellonian University, Krakow, Poland; 64 Institute of Nuclear Physics, Polish Academy of Sciences, Kraków, Poland; 65 Physics Department, Southern Methodist University, Dallas, TX USA; 66 Physics Department, University of Texas at Dallas, Richardson, TX USA; 67 DESY, Hamburg and Zeuthen, Germany; 68 Lehrstuhl für Experimentelle Physik IV, Technische Universität Dortmund, Dortmund, Germany; 69 Institut für Kern- und Teilchenphysik, Technische Universität Dresden, Dresden, Germany; 70 Department of Physics, Duke University, Durham, NC USA; 71 SUPA - School of Physics and Astronomy, University of Edinburgh, Edinburgh, UK; 72 INFN e Laboratori Nazionali di Frascati, Frascati, Italy; 73 Fakultät für Mathematik und Physik, Albert-Ludwigs-Universität, Freiburg, Germany; 74 Departement de Physique Nucleaire et Corpusculaire, Université de Genève, Geneva, Switzerland; 75 INFN Sezione di Genova, Genova, Italy; 76 Dipartimento di Fisica, Università di Genova, Genoa, Italy; 77 E. Andronikashvili Institute of Physics, Iv. Javakhishvili Tbilisi State University, Tbilisi, Georgia; 78 High Energy Physics Institute, Tbilisi State University, Tbilisi, Georgia; 79 II Physikalisches Institut, Justus-Liebig-Universität Giessen, Giessen, Germany; 80 SUPA - School of Physics and Astronomy, University of Glasgow, Glasgow, UK; 81 II Physikalisches Institut, Georg-August-Universität, Göttingen, Germany; 82 Laboratoire de Physique Subatomique et de Cosmologie, Université Grenoble-Alpes, CNRS/IN2P3, Grenoble, France; 83 Laboratory for Particle Physics and Cosmology, Harvard University, Cambridge, MA USA; 84 Kirchhoff-Institut für Physik, Ruprecht-Karls-Universität Heidelberg, Heidelberg, Germany; 85 Physikalisches Institut, Ruprecht-Karls-Universität Heidelberg, Heidelberg, Germany; 86 Faculty of Applied Information Science, Hiroshima Institute of Technology, Hiroshima, Japan; 87 Department of Physics, The Chinese University of Hong Kong, Shatin, N.T. Hong Kong; 88 Department of Physics, The University of Hong Kong, Hong Kong, China; 89 Department of Physics, Institute for Advanced Study, The Hong Kong University of Science and Technology, Clear Water Bay, Kowloon, Hong Kong, China; 90 Department of Physics, National Tsing Hua University, Taiwan, Taiwan; 91 Department of Physics, Indiana University, Bloomington, IN USA; 92 Institut für Astro- und Teilchenphysik, Leopold-Franzens-Universität, Innsbruck, Austria; 93 University of Iowa, Iowa City, IA USA; 94 Department of Physics and Astronomy, Iowa State University, Ames, IA USA; 95 Joint Institute for Nuclear Research, JINR Dubna, Dubna, Russia; 96 KEK, High Energy Accelerator Research Organization, Tsukuba, Japan; 97 Graduate School of Science, Kobe University, Kobe, Japan; 98 Faculty of Science, Kyoto University, Kyoto, Japan; 99 Kyoto University of Education, Kyoto, Japan; 100 Research Center for Advanced Particle Physics and Department of Physics, Kyushu University, Fukuoka, Japan; 101 Instituto de Física La Plata, Universidad Nacional de La Plata and CONICET, La Plata, Argentina; 102 Physics Department, Lancaster University, Lancaster, UK; 103 INFN Sezione di Lecce, Lecce, Italy; 104 Dipartimento di Matematica e Fisica, Università del Salento, Lecce, Italy; 105 Oliver Lodge Laboratory, University of Liverpool, Liverpool, UK; 106 Department of Experimental Particle Physics, Jožef Stefan Institute and Department of Physics, University of Ljubljana, Ljubljana, Slovenia; 107 School of Physics and Astronomy, Queen Mary University of London, London, UK; 108 Department of Physics, Royal Holloway University of London, Surrey, UK; 109 Department of Physics and Astronomy, University College London, London, UK; 110 Louisiana Tech University, Ruston, LA USA; 111 Laboratoire de Physique Nucléaire et de Hautes Energies, UPMC and Université Paris-Diderot and CNRS/IN2P3, Paris, France; 112 Fysiska institutionen, Lunds universitet, Lund, Sweden; 113 Departamento de Fisica Teorica C-15, Universidad Autonoma de Madrid, Madrid, Spain; 114 Institut für Physik, Universität Mainz, Mainz, Germany; 115 School of Physics and Astronomy, University of Manchester, Manchester, UK; 116 CPPM, Aix-Marseille Université and CNRS/IN2P3, Marseille, France; 117 Department of Physics, University of Massachusetts, Amherst, MA USA; 118 Department of Physics, McGill University, Montreal, QC Canada; 119 School of Physics, University of Melbourne, Victoria, Australia; 120 Department of Physics, The University of Michigan, Ann Arbor, MI USA; 121 Department of Physics and Astronomy, Michigan State University, East Lansing, MI USA; 122 INFN Sezione di Milano, Milano, Italy; 123 Dipartimento di Fisica, Università di Milano, Milan, Italy; 124 B.I. Stepanov Institute of Physics, National Academy of Sciences of Belarus, Minsk, Republic of Belarus; 125 Research Institute for Nuclear Problems of Byelorussian State University, Minsk, Republic of Belarus; 126 Group of Particle Physics, University of Montreal, Montreal, QC Canada; 127 P.N. Lebedev Physical Institute of the Russian Academy of Sciences, Moscow, Russia; 128 Institute for Theoretical and Experimental Physics (ITEP), Moscow, Russia; 129 National Research Nuclear University MEPhI, Moscow, Russia; 130 D.V. Skobeltsyn Institute of Nuclear Physics, M.V.Lomonosov Moscow State University, Moscow, Russia; 131 Fakultät für Physik, Ludwig-Maximilians-Universität München, München, Germany; 132 Max-Planck-Institut für Physik (Werner-Heisenberg-Institut), München, Germany; 133 Nagasaki Institute of Applied Science, Nagasaki, Japan; 134 Graduate School of Science and Kobayashi-Maskawa Institute, Nagoya University, Nagoya, Japan; 135 INFN Sezione di Napoli, Napoli, Italy; 136 Dipartimento di Fisica, Università di Napoli, Napoli, Italy; 137 Department of Physics and Astronomy, University of New Mexico, Albuquerque, NM USA; 138 Institute for Mathematics, Astrophysics and Particle Physics, Radboud University Nijmegen/Nikhef, Nijmegen, The Netherlands; 139 Nikhef National Institute for Subatomic Physics, University of Amsterdam, Amsterdam, The Netherlands; 140 Department of Physics, Northern Illinois University, DeKalb, IL USA; 141 Budker Institute of Nuclear Physics, SB RAS, Novosibirsk, Russia; 142 Department of Physics, New York University, New York, NY USA; 143 Ohio State University, Columbus, OH USA; 144 Faculty of Science, Okayama University, Okayama, Japan; 145 Homer L. Dodge Department of Physics and Astronomy, University of Oklahoma, Norman, OK USA; 146 Department of Physics, Oklahoma State University, Stillwater, OK USA; 147 Palacký University, RCPTM, Olomouc, Czech Republic; 148 Center for High Energy Physics, University of Oregon, Eugene, OR USA; 149 LAL, Univ. Paris-Sud, CNRS/IN2P3, Université Paris-Saclay, Orsay, France; 150 Graduate School of Science, Osaka University, Osaka, Japan; 151 Department of Physics, University of Oslo, Oslo, Norway; 152 Department of Physics, Oxford University, Oxford, UK; 153 INFN Sezione di Pavia, Pavia, Italy; 154 Dipartimento di Fisica, Università di Pavia, Pavia, Italy; 155 Department of Physics, University of Pennsylvania, Philadelphia, PA USA; 156 National Research Centre “Kurchatov Institute” B.P. Konstantinov Petersburg Nuclear Physics Institute, St. Petersburg, Russia; 157 INFN Sezione di Pisa, Pisa, Italy; 158 Dipartimento di Fisica E. Fermi, Università di Pisa, Pisa, Italy; 159 Department of Physics and Astronomy, University of Pittsburgh, Pittsburgh, PA USA; 160 Laboratório de Instrumentação e Física Experimental de Partículas - LIP, Lisbon, Portugal; 161 Faculdade de Ciências, Universidade de Lisboa, Lisboa, Portugal; 162 Department of Physics, University of Coimbra, Coimbra, Portugal; 163 Centro de Física Nuclear da Universidade de Lisboa, Lisboa, Portugal; 164 Departamento de Fisica, Universidade do Minho, Braga, Portugal; 165 Departamento de Fisica Teorica y del Cosmos, Universidad de Granada, Granada, Spain; 166 Dep Fisica and CEFITEC of Faculdade de Ciencias e Tecnologia, Universidade Nova de Lisboa, Caparica, Portugal; 167 Institute of Physics, Academy of Sciences of the Czech Republic, Praha, Czech Republic; 168 Czech Technical University in Prague, Praha, Czech Republic; 169 Faculty of Mathematics and Physics, Charles University, Prague, Czech Republic; 170 State Research Center Institute for High Energy Physics (Protvino), NRC KI, Protvino, Russia; 171 Particle Physics Department, Rutherford Appleton Laboratory, Didcot, UK; 172 INFN Sezione di Roma, Roma, Italy; 173 Dipartimento di Fisica, Sapienza Università di Roma, Roma, Italy; 174 INFN Sezione di Roma Tor Vergata, Roma, Italy; 175 Dipartimento di Fisica, Università di Roma Tor Vergata, Roma, Italy; 176 INFN Sezione di Roma Tre, Roma, Italy; 177 Dipartimento di Matematica e Fisica, Università Roma Tre, Roma, Italy; 178 Faculté des Sciences Ain Chock, Réseau Universitaire de Physique des Hautes Energies-Université Hassan II, Casablanca, Morocco; 179 Centre National de l’Energie des Sciences Techniques Nucleaires, Rabat, Morocco; 180 Faculté des Sciences Semlalia, Université Cadi Ayyad, LPHEA-Marrakech, Marrakech, Morocco; 181 Faculté des Sciences, Université Mohamed Premier and LPTPM, Oujda, Morocco; 182 Faculté des Sciences, Université Mohammed V, Rabat, Morocco; 183 DSM/IRFU (Institut de Recherches sur les Lois Fondamentales de l’Univers), CEA Saclay (Commissariat à l’Energie Atomique et aux Energies Alternatives), Gif-sur-Yvette, France; 184 Santa Cruz Institute for Particle Physics, University of California Santa Cruz, Santa Cruz, CA USA; 185 Department of Physics, University of Washington, Seattle, WA USA; 186 Department of Physics and Astronomy, University of Sheffield, Sheffield, UK; 187 Department of Physics, Shinshu University, Nagano, Japan; 188 Department Physik, Universität Siegen, Siegen, Germany; 189 Department of Physics, Simon Fraser University, Burnaby, BC Canada; 190 SLAC National Accelerator Laboratory, Stanford, CA USA; 191 Faculty of Mathematics, Physics and Informatics, Comenius University, Bratislava, Slovak Republic; 192 Department of Subnuclear Physics, Institute of Experimental Physics of the Slovak Academy of Sciences, Kosice, Slovak Republic; 193 Department of Physics, University of Cape Town, Cape Town, South Africa; 194 Department of Physics, University of Johannesburg, Johannesburg, South Africa; 195 School of Physics, University of the Witwatersrand, Johannesburg, South Africa; 196 Department of Physics, Stockholm University, Stockholm, Sweden; 197 The Oskar Klein Centre, Stockholm, Sweden; 198 Physics Department, Royal Institute of Technology, Stockholm, Sweden; 199 Departments of Physics and Astronomy and Chemistry, Stony Brook University, Stony Brook, NY USA; 200 Department of Physics and Astronomy, University of Sussex, Brighton, UK; 201 School of Physics, University of Sydney, Sydney, Australia; 202 Institute of Physics, Academia Sinica, Taipei, Taiwan; 203 Department of Physics, Technion: Israel Institute of Technology, Haifa, Israel; 204 Raymond and Beverly Sackler School of Physics and Astronomy, Tel Aviv University, Tel Aviv, Israel; 205 Department of Physics, Aristotle University of Thessaloniki, Thessaloniki, Greece; 206 International Center for Elementary Particle Physics and Department of Physics, The University of Tokyo, Tokyo, Japan; 207 Graduate School of Science and Technology, Tokyo Metropolitan University, Tokyo, Japan; 208 Department of Physics, Tokyo Institute of Technology, Tokyo, Japan; 209 Tomsk State University, Tomsk, Russia; 210 Department of Physics, University of Toronto, Toronto, ON Canada; 211 INFN-TIFPA, Trento, Italy; 212 University of Trento, Trento, Italy; 213 TRIUMF, Vancouver, BC Canada; 214 Department of Physics and Astronomy, York University, Toronto, ON Canada; 215 Faculty of Pure and Applied Sciences, and Center for Integrated Research in Fundamental Science and Engineering, University of Tsukuba, Tsukuba, Japan; 216 Department of Physics and Astronomy, Tufts University, Medford, MA USA; 217 Department of Physics and Astronomy, University of California Irvine, Irvine, CA USA; 218 INFN Gruppo Collegato di Udine, Sezione di Trieste, Udine, Italy; 219 ICTP, Trieste, Italy; 220 Dipartimento di Chimica, Fisica e Ambiente, Università di Udine, Udine, Italy; 221 Department of Physics and Astronomy, University of Uppsala, Uppsala, Sweden; 222 Department of Physics, University of Illinois, Urbana, IL USA; 223 Instituto de Fisica Corpuscular (IFIC), Centro Mixto Universidad de Valencia-CSIC, Valencia, Spain; 224 Department of Physics, University of British Columbia, Vancouver, BC Canada; 225 Department of Physics and Astronomy, University of Victoria, Victoria, BC Canada; 226 Department of Physics, University of Warwick, Coventry, UK; 227 Waseda University, Tokyo, Japan; 228 Department of Particle Physics, The Weizmann Institute of Science, Rehovot, Israel; 229 Department of Physics, University of Wisconsin, Madison, WI USA; 230 Fakultät für Physik und Astronomie, Julius-Maximilians-Universität, Würzburg, Germany; 231 Fakultät für Mathematik und Naturwissenschaften, Fachgruppe Physik, Bergische Universität Wuppertal, Wuppertal, Germany; 232 Department of Physics, Yale University, New Haven, CT USA; 233 Yerevan Physics Institute, Yerevan, Armenia; 234 Centre de Calcul de l’Institut National de Physique Nucléaire et de Physique des Particules (IN2P3), Villeurbanne, France; 235 Academia Sinica Grid Computing, Institute of Physics, Academia Sinica, Taipei, Taiwan; 236 CERN, 1211 Geneva 23, Switzerland

## Abstract

A search for heavy resonances decaying into a pair of $Z$ bosons leading to $\ell ^+\ell ^-\ell ^+\ell ^-$ and $\ell ^+\ell ^-\nu \bar{\nu }$ final states, where $\ell$ stands for either an electron or a muon, is presented. The search uses proton–proton collision data at a centre-of-mass energy of 13 $\text {TeV}$ corresponding to an integrated luminosity of 36.1 $\hbox {fb}^{-1}$ collected with the ATLAS detector during 2015 and 2016 at the Large Hadron Collider. Different mass ranges for the hypothetical resonances are considered, depending on the final state and model. The different ranges span between 200 and 2000 $\text {GeV}$. The results are interpreted as upper limits on the production cross section of a spin-0 or spin-2 resonance. The upper limits for the spin-0 resonance are translated to exclusion contours in the context of Type-I and Type-II two-Higgs-doublet models, while those for the spin-2 resonance are used to constrain the Randall–Sundrum model with an extra dimension giving rise to spin-2 graviton excitations.

## Introduction

In 2012, the ATLAS and CMS Collaborations at the LHC discovered a new particle [1, 2], an important milestone in the understanding of the mechanism of electroweak (EW) symmetry breaking [3–5]. Both experiments have confirmed that the spin, parity and couplings of the new particle are consistent with those predicted for the Standard Model (SM) Higgs boson [6–8] (denoted by $h$ throughout this paper). They measured its mass to be $m_{h} = 125.09 \pm 0.21 \mathrm {(stat)} \pm 0.11 \mathrm {(syst)}$ $\text {GeV}$[9] and reported recently on a combination of measurements of its couplings to other SM particles [10].

One important question is whether the newly discovered particle is part of an extended scalar sector as postulated by various extensions to the Standard Model such as the two-Higgs-doublet model (2HDM) [11]. These extensions predict additional Higgs bosons, motivating searches in an extended range of mass.

This paper reports on two searches for a heavy resonance decaying into two SM *Z* bosons, encompassing the final states $ZZ\!\rightarrow \!\ell ^+\ell ^-\ell ^+\ell ^-$ and $ZZ\!\rightarrow \!\ell ^+\ell ^-\nu \bar{\nu }$ where $\ell$ stands for either an electron or a muon and $\nu$ stands for all three neutrino flavours. These final states are referred to as $\ell ^+\ell ^-\ell ^+\ell ^-$ and $\ell ^+\ell ^-\nu \bar{\nu }$ respectively.

It is assumed that an additional Higgs boson (denoted as $$H$$ throughout this paper) would be produced predominantly via gluon–gluon fusion (ggF) and vector-boson fusion (VBF) processes, but that the ratio of the two production mechanisms is unknown in the absence of a specific model. For this reason, the results are interpreted separately for the ggF and VBF production modes, with events being classified into ggF- and VBF-enriched categories in both final states, as discussed in Sects. 5 and 6. With good mass resolution and a high signal-to-background ratio, the $$\ell ^+\ell ^-\ell ^+\ell ^-$$ final state is well suited to a search for a narrow resonance with mass $$m_{H} $$ between $$200~\text {GeV}$$ and $$1200~\text {GeV}$$. The $$\ell ^+\ell ^-\nu \bar{\nu }$$ search covers the $$300~\text {GeV}<m_{H} <1400~\text {GeV}$$ range and dominates at high masses due to its larger branching ratio.

These searches look for an excess in distributions of the four-lepton invariant mass, $m_{4\ell }$, for the $\ell ^+\ell ^-\ell ^+\ell ^-$ final state, and the transverse invariant mass, $m_{\mathrm {T}}$, for the $\ell ^+\ell ^-\nu \bar{\nu }$ final state, as the escaping neutrinos do not allow the full reconstruction of the final state. The transverse invariant mass is defined as:

$$\begin{aligned} m_{\mathrm {T}} \equiv \sqrt{ \left[ {\sqrt{m_{Z} ^{2} + \left( p_{\text {T}} ^{\ell \ell }\right) ^2} + \sqrt{m_{Z} ^{2} + \left( E_{\text {T}}^{\text {miss}} \right) ^2}}\, \right] ^2 - \left|\vec {p_{\text {T}}}^{\ell \ell } + \vec {E}_{\text {T}}^{\text {miss}} \right|^2 }, \end{aligned}$$

where $m_{Z}$ is the mass of the $Z$ boson, $p_{\text {T}} ^{\ell \ell }$ is the transverse momentum of the lepton pair and $\vec {E}_{\text {T}}^{\text {miss}}$ is the missing transverse momentum, with magnitude $E_{\text {T}}^{\text {miss}}$. In the absence of such an excess, limits on the production rate of different signal hypotheses are obtained from a simultaneous likelihood fit to the two mass distributions. The first hypothesis is the ggF and VBF production of a heavy Higgs boson (spin-0 resonance) under the narrow-width approximation (NWA). The upper limits on the production rate of a heavy Higgs boson are then translated into exclusion contours in the context of the two-Higgs-doublet model. As several theoretical models favour non-negligible natural widths, large-width assumption (LWA) models, assuming widths of 1%, 5% and 10% of the resonance mass, are also studied. The interference between the heavy scalar and the SM Higgs boson as well as between the heavy scalar and the $gg \rightarrow ZZ$ continuum background are taken into account in this study. Limits are also set on the Randall–Sundrum (RS) model [12, 13] with a warped extra dimension giving rise to a spin-2 Kaluza–Klein (KK) excitation of the graviton $G_{\mathrm {KK}}$.

Other searches for diboson resonances decaying into *WW* or *ZZ* or *WZ* have been performed by ATLAS [14–16] and CMS [17–19].

With a significant increase in integrated luminosity and an improved discovery potential from the higher parton luminosities [20] at a centre-of-mass energy of $\sqrt{s}$ = 13 $\text {TeV}$ as compared to $\sqrt{s}$ = 8 $\text {TeV}$, the results of this paper improve upon previous results published by the ATLAS Collaboration from a search for an additional heavy Higgs boson [21]. Results of a similar search from the data collected at the LHC with $\sqrt{s}$ = 8 $\text {TeV}$ have also been reported by the CMS Collaboration [22].

## ATLAS detector

The ATLAS experiment is described in detail in Ref. [23]. ATLAS is a multi-purpose detector with a forward–backward symmetric cylindrical geometry and a solid-angle^1^ coverage of nearly 4$\pi$. The inner tracking detector (ID), covering the region $|\eta |<$ 2.5, consists of a silicon pixel detector, a silicon microstrip detector and a transition-radiation tracker. The innermost layer of the pixel detector, the insertable B-layer (IBL) [24], was installed between Run 1 and Run 2 of the LHC. The inner detector is surrounded by a thin superconducting solenoid providing a $2\,\text {T}$ magnetic field, and by a finely segmented lead/liquid-argon (LAr) electromagnetic calorimeter covering the region $|\eta |<$ 3.2. A steel/scintillator-tile hadronic calorimeter provides coverage in the central region $|\eta |<$ 1.7. The end-cap and forward regions, covering the pseudorapidity range 1.5 $< |\eta |<$ 4.9, are instrumented with electromagnetic and hadronic LAr calorimeters, with steel, copper or tungsten as the absorber material. A muon spectrometer (MS) system incorporating large superconducting toroidal air-core magnets surrounds the calorimeters. Three layers of precision wire chambers provide muon tracking in the range $|\eta |<$ 2.7, while dedicated fast chambers are used for triggering in the region $|\eta |<$ 2.4. The trigger system, composed of two stages, was upgraded [25] before Run 2. The first stage, implemented with custom hardware, uses information from calorimeters and muon chambers to reduce the event rate from about 40 MHz to a maximum of 100 kHz. The second stage, called the high-level trigger (HLT), reduces the data acquisition rate to about 1 kHz on average. The HLT is software-based and runs reconstruction algorithms similar to those used in the offline reconstruction.

## Data and Monte Carlo samples

The proton–proton (*pp*) collision data used in these searches were collected by the ATLAS detector at a centre-of-mass energy of 13 $\text {TeV}$ with a 25 ns bunch-spacing configuration during 2015 and 2016. The data are subjected to quality requirements: if any relevant detector component is not operating correctly during a period in which an event is recorded, the event is rejected. After these quality requirements, the total accumulated data sample corresponds to an integrated luminosity of 36.1 $\hbox {fb}^{-1}$.

Simulated events are used to determine the signal acceptance and some of the background contributions to these searches. The particle-level events produced by each Monte Carlo (MC) event generator were processed through the ATLAS detector simulation [26] within the $\textsc {Geant}~4$ framework [27]. Additional inelastic *pp* interactions (pile-up) were overlaid on the simulated signal and background events. The MC event generator used for this is Pythia 8.186 [28] with the A2 set of tuned parameters [29] and the MSTW2008LO [30] parton distribution functions (PDF) set. The simulated events are weighted to reproduce the observed distribution of the mean number of interactions per bunch crossing in data (pile-up reweighting). The properties of the bottom and charm hadron decays were simulated by the EvtGen v1.2.0 program [31].

Heavy spin-0 resonance production was simulated using the Powheg-Box v2 [32] MC event generator. Gluon–gluon fusion and vector-boson fusion production modes were calculated separately with matrix elements up to next-to-leading order (NLO) in QCD. Powheg-Box was interfaced to Pythia 8.212 [33] for parton showering and hadronisation, and for decaying the Higgs boson into the $H\!\rightarrow \!ZZ\!\rightarrow \!\ell ^+\ell ^-\ell ^+\ell ^-$ or $H\!\rightarrow \!ZZ\!\rightarrow \!\ell ^+\ell ^-\nu \bar{\nu }$ final states. The CT10 PDF set [34] was used for the hard process. Events from ggF and VBF production were generated in the $300~\text {GeV}< m_{H} < 1600$ $\text {GeV}$ mass range under the NWA, using a step of 100 (200) $\text {GeV}$ up to (above) 1000 $\text {GeV}$ in mass. For the $\ell ^+\ell ^-\ell ^+\ell ^-$ final state, due to the sensitivity of the analysis at lower masses, events were also generated for $m_{H} = 200$ $\text {GeV}$.

In addition, events from ggF production with a boson width of 5, 10 and 15% of the scalar mass $m_{H}$ were generated with MadGraph5_aMC@NLO v2.3.2 [35] interfaced to Pythia 8.210 for parton showering and hadronisation for both final states. For the $\ell ^+\ell ^-\ell ^+\ell ^-$ final state, the $m_{4\ell }$ distribution is parameterised analytically as described in Sect. 5.3, and the samples with a width of 15% of $m_{H}$ are used to validate the parameterisation. For the $\ell ^+\ell ^-\nu \bar{\nu }$ final state, a reweighting procedure as described in Sect. 6.3 is used on fully simulated events to obtain the reconstructed $m_{\mathrm {T}}$ distribution at any value of mass and width tested. To have a better description of the jet multiplicity, MadGraph5_aMC@NLO was also used to generate events for the process $pp \rightarrow H$ + $\ge$ 2 jets at NLO QCD accuracy with the FxFx merging scheme [36].

The fraction of the ggF events that enter into the VBF-enriched category is estimated from the MadGraph5_aMC@NLO simulation.

Spin-2 Kaluza–Klein gravitons from the Bulk Randall–Sundrum model [37] were generated with MadGraph5_aMC@NLO at leading order (LO) in QCD. The dimensionless coupling $k/{\bar{M}_\mathrm {Pl}}$, where $\bar{M}_\mathrm {Pl} = M_\mathrm {Pl}/\sqrt{8\pi }$ is the reduced Planck scale and *k* is the curvature scale of the extra dimension, is set to 1. In this configuration, the width of the resonance is expected to be $\sim 6\%$ of its mass.

Mass points between 600 $\text {GeV}$ and 2 $\text {TeV}$ with 200 $\text {GeV}$ spacing were generated for the $\ell ^+\ell ^-\nu \bar{\nu }$ final state. These samples were processed through a fast detector simulation [26] that uses a parameterisation of the response of electromagnetic and hadronic calorimeters [38], while the response of the ID and MS detectors is fully simulated.

The $q\bar{q} \rightarrow ZZ$ background for the $\ell ^+\ell ^-\nu \bar{\nu }$ final state was simulated by the Powheg-Box v2 event generator [32] and interfaced to Pythia 8.186 [28] for parton showering and hadronisation. The CT10nlo PDF set [34] was used for hard-scattering processes. Next-to-next-to-leading-order (NNLO) QCD and NLO EW corrections are included [39–41] as a function of the invariant mass $m_{ZZ}$ of the $ZZ$ system. For the $\ell ^+\ell ^-\ell ^+\ell ^-$ final state, this background was simulated with the Sherpa v2.2.1 [42–44] event generator, with the NNPDF3.0 NNLO PDF set [45] for the hard-scattering process. NLO accuracy is achieved in the matrix-element calculation for 0- and 1-jet final states and LO accuracy for 2- and 3-jet final states. The merging with the Sherpa parton shower [46] was performed using the MePs@NLO prescription [47].

NLO EW corrections were applied as a function of $m_{ZZ}$ [41, 48]. In addition, Sherpa v2.2.1 was used for the $\ell ^+\ell ^-\nu \bar{\nu }$ final state to scale the fraction of events in the VBF-enriched category obtained from Powheg-Box simulation, because the Sherpa event generator calculates matrix elements up to one parton at NLO and up to three partons at LO. The EW production of a $ZZ$ pair and two additional jets via vector-boson scattering up to $O(\alpha _\mathrm {EW}^6)$ was generated using Sherpa, where the process $ZZZ\rightarrow 4\ell qq$ is also taken into account.

The $gg \rightarrow ZZ$ production was modelled by Sherpa v2.1.1 at LO in QCD for the $\ell ^+\ell ^-\ell ^+\ell ^-$ final state and by gg2VV [49] for the $\ell ^+\ell ^-\nu \bar{\nu }$ final state, both including the off-shell $h$ boson contribution and the interference between the $h$ and $ZZ$ backgrounds. The K-factor accounting for higher-order QCD effects for the $gg \rightarrow ZZ$ continuum production was calculated for massless quark loops [50–52] in the heavy-top-quark approximation [53], including the $gg\rightarrow H^{*} \rightarrow ZZ$ process [54]. Based on these studies, a constant K-factor of 1.7 is used, and a relative uncertainty of 60% is assigned to the normalisation in both searches.

The $WW$ and $WZ$ diboson events were simulated by Powheg-Box, using the CT10nlo PDF set and Pythia 8.186 for parton showering and hadronisation. The production cross section of these samples is predicted at NLO in QCD.

Events containing a single *Z* boson with associated jets were simulated using the Sherpa v2.2.1 event generator. Matrix elements were calculated for up to two partons at NLO and four partons at LO using the Comix [43] and OpenLoops [44] matrix-element generators and merged with the Sherpa parton shower [46] using the ME+PS@NLO prescription [47]. The NNPDF3.0 NNLO PDF set was used in conjunction with dedicated parton-shower tuning developed by the Sherpa authors. The $Z$ + jets events are normalised using the NNLO cross sections [55].

The triboson backgrounds *ZZZ*, *WZZ*, and *WWZ* with fully leptonic decays and at least four prompt charged leptons were modelled using Sherpa v2.1.1. For the fully leptonic $t\bar{t}+Z$ background, with four prompt leptons originating from the decays of the top quarks and *Z* boson, MadGraph5_aMC@NLO was used. The $t\bar{t}$ background, as well as the single-top and *Wt* production, were modelled using Powheg-Box v2 interfaced to Pythia 6.428 [56] with the Perugia 2012 [57] set of tuned parameters for parton showering and hadronisation, to PHOTOS [58] for QED radiative corrections and to Tauola [59, 60] for the simulation of $\tau$-lepton decays.

In order to study the interference treatment for the LWA case, samples containing the $gg \rightarrow ZZ$ continuum background (*B*) as well as its interference (*I*) with a hypothetical heavy scalar (*S*) were used and are referred to as *SBI* samples hereafter. In the $\ell ^+\ell ^-\ell ^+\ell ^-$ final state the MCFM NLO event generator [61], interfaced to Pythia 8.212, was used to produce *SBI* samples where the width of the heavy scalar is set to 15% of its mass, for masses of 200, 300, 400, 500, 600, 800, 1000, 1200 and 1400 $\text {GeV}$. Background-only samples were also generated with the MCFM event generator, and are used to extract the signal-plus-interference term (*SI*) by subtracting them from the aforementioned *SBI* samples. For the $\ell ^+\ell ^-\nu \bar{\nu }$ final state, the *SBI* samples were generated with the gg2VV event generator. The samples include signal events with a scalar mass of 400, 700, 900, 1200 and 1500 $\text {GeV}$.

## Event reconstruction

Electrons are reconstructed using information from the ID and the electromagnetic calorimeter [62]. Electron candidates are clusters of energy deposits associated with ID tracks, where the final track–cluster matching is performed after the tracks have been fitted with a Gaussian-sum filter (GSF) to account for bremsstrahlung energy losses. Background rejection relies on the longitudinal and transverse shapes of the electromagnetic showers in the calorimeters, track–cluster matching and properties of tracks in the ID. All of this information, except for that related to track hits, is combined into a likelihood discriminant.

The selection used combines the likelihood with the number of track hits and defines two working points (WP) which are used in the analyses presented here. The $\ell ^+\ell ^-\ell ^+\ell ^-$ analysis uses a “loose” WP, with an efficiency ranging from 90% for transverse momentum $p_{\text {T}} =20~\text {GeV}$ to 96% for $p_{\text {T}} >60~\text {GeV}$. A “medium” WP was chosen for the $\ell ^+\ell ^-\nu \bar{\nu }$ analysis with an efficiency increasing from 82% at $p_{\text {T}} =20~\text {GeV}$ to 93% for $p_{\text {T}} >60~\text {GeV}$. The electron’s transverse momentum is computed from the cluster energy and the track direction at the interaction point.

Muons are formed from tracks reconstructed in the ID and MS, and their identification is primarily based on the presence of the track or track segment in the MS [63]. If a complete track is present in both the ID and the MS, a combined muon track is formed by a global fit using the hit information from both the ID and MS detectors (combined muon), otherwise the momentum is measured using the ID, and the MS track segment serves as identification (segment-tagged muon). The segment-tagged muon is limited to the centre of the barrel region ($$|\eta | <0.1$$) which has reduced MS geometrical coverage. Furthermore, in this central region an ID track with $$p_{\text {T}}$$ $$ > 15$$ $$\text {GeV}$$ is identified as a muon if its calorimetric energy deposition is consistent with a minimum-ionising particle (calorimeter-tagged muon). In the forward region ($$2.5< |\eta | < 2.7$$) with limited or no ID coverage, the MS track is either used alone (stand-alone muon) or combined with silicon hits, if found in the forward ID (combined muon). The ID tracks associated with the muons are required to have a minimum number of associated hits in each of the ID subdetectors to ensure good track reconstruction. The stand-alone muon candidates are required to have hits in each of the three MS stations they traverse. A “loose” muon identification WP, which uses all muon types and has an efficiency of 98.5%, is adopted by the $$\ell ^+\ell ^-\ell ^+\ell ^- $$ analysis. For the $$\ell ^+\ell ^-\nu \bar{\nu } $$ analysis a “medium” WP is used, which only includes combined muons and has an efficiency of 97%.

Jets are reconstructed using the anti-$k_t$ algorithm [64] with a radius parameter *R* = 0.4 implemented in the FastJet package [65], and positive-energy clusters of calorimeter cells as input. The algorithm suppresses noise and pile-up by keeping only cells with a significant energy deposit and their neighbouring cells. Jets are calibrated using a dedicated scheme designed to adjust, on average, the energy measured in the calorimeter to that of the true jet energy [66]. The jets used in this analysis are required to satisfy $p_{\text {T}} > 20~\text {GeV}$ and $|\eta |<4.5$. To reduce the number of jet candidates originating from pile-up vertices, an additional requirement that uses the track and vertex information inside a jet is imposed on jets with $p_{\text {T}} < 60~\text {GeV}$ and $|\eta |<2.4$ [67].

Jets containing *b*-hadrons, referred to as *b*-jets, are identified by the long lifetime, high mass and decay multiplicity of *b*-hadrons, as well as the hard *b*-quark fragmentation function. The $\ell ^+\ell ^-\nu \bar{\nu }$ analysis identifies *b*-jets of $p_{\text {T}} > 20~\text {GeV}$ and $|\eta |<2.5$ using an algorithm that achieves an identification efficiency of about 85% in simulated $t\bar{t}$ events, with a rejection factor for light-flavour jets of about 33 [68, 69].

Selected events are required to have at least one vertex with two associated tracks with $p_{\text {T}}$ > 400 $\text {MeV}$, and the primary vertex is chosen to be the vertex reconstructed with the largest $\sum p_{\text {T}} ^2$. As lepton and jet candidates can be reconstructed from the same detector information, a procedure to resolve overlap ambiguities is applied. If an electron and a muon share the same ID track, the muon is selected unless it is calorimeter-tagged and does not have a MS track, or is a segment-tagged muon, in which case the electron is selected. Reconstructed jets which overlap with electrons (muons) in a cone of size $\Delta R\equiv \sqrt{(\Delta \eta )^2 + (\Delta \phi )^2}=$ 0.2 (0.1) are removed.

The missing transverse momentum $\vec {E}_{\text {T}}^{\text {miss}}$, which accounts for the imbalance of visible momenta in the plane transverse to the beam axis, is computed as the negative vector sum of the transverse momenta of all identified electrons, muons and jets, as well as a “soft term”, accounting for unclassified soft tracks and energy clusters in the calorimeters [70]. This analysis uses a track-based soft term, which is built by combining the information provided by the ID and the calorimeter, in order to minimise the effect of pile-up which degrades the $E_{\text {T}}^{\text {miss}}$ resolution. The soft term is computed using the momenta of the tracks associated with the primary vertex, while the jet and electron momenta are computed at the calorimeter level to allow the inclusion of neutral particles. Jet–muon overlap is accounted for in the $E_{\text {T}}^{\text {miss}}$ calculation. This corrects for fake jets due to pile-up close to muons and double-counted jets from muon energy losses.

## $H\!\rightarrow \!ZZ\!\rightarrow \!\ell ^+\ell ^-\ell ^+\ell ^-$ event selection and background estimation

### Event selection

Four-lepton events are selected and initially classified according to the lepton flavours: $4\mu$, $2e2\mu$, 4*e*, called “channels” hereafter. They are selected with single-lepton, dilepton and trilepton triggers, with the dilepton and trilepton ones including electron(s)–muon(s) triggers. Single-electron triggers apply “medium” or “tight” likelihood identification, whereas multi-electron triggers apply “loose” or “medium” identification. For the bulk of the data, recorded in 2016, the lowest $p_{\text {T}}$ threshold for the single-electron (muon) triggers used is set to 26 (26) $\text {GeV}$, for the dielectron (dimuon) triggers to 15 (10) $\text {GeV}$ and for the trielectron (trimuon) triggers to 12 (6) $\text {GeV}$. For the data collected in 2015, the instantaneous luminosity was lower so the trigger thresholds were lower; this increases the signal efficiency by less than 1%. Globally, the trigger efficiency for signal events passing the final selection requirements is about 98%.

In each channel, four-lepton candidates are formed by selecting a lepton-quadruplet made out of two same-flavour, opposite-sign lepton pairs, selected as described in Sect. 4. Each electron (muon) must satisfy $p_{\text {T}} > 7$ (5) $\text {GeV}$ and be measured in the pseudorapidity range of $\left| \eta \right| <2.47$ (2.7). The highest-$p_{\text {T}}$ lepton in the quadruplet must satisfy $p_{\text {T}}$ $> 20$ $\text {GeV}$, and the second (third) lepton in $p_{\text {T}}$ order must satisfy $p_{\text {T}}$ $> 15$ $\text {GeV}$ (10 $\text {GeV}$). In the case of muons, at most one calorimeter-tagged, segment-tagged or stand-alone ($2.5< |\eta | < 2.7$) muon is allowed per quadruplet.

If there is ambiguity in assigning leptons to a pair, only one quadruplet per channel is selected by keeping the quadruplet with the lepton pairs closest (leading pair) and second closest (subleading pair) to the *Z* boson mass, with invariant masses referred to as $m_{12}$ and $m_{34}$ respectively. In the selected quadruplet, $m_{12}$ is required to be $50~\text {GeV}< m_{12} < 106~\text {GeV}$, while $m_{34}$ is required to be less than 115 $\text {GeV}$ and greater than a threshold that is 12 $\text {GeV}$ for $m_{4\ell } \le 140~\text {GeV}$, rises linearly from 12 $\text {GeV}$ to 50 $\text {GeV}$ with $m_{4\ell }$ in the interval of [140 $\text {GeV}$, 190 $\text {GeV}$] and is fixed to 50 $\text {GeV}$ for $m_{4\ell } > 190~\text {GeV}$.

**Table 1:** Signal acceptance for the $\ell ^+\ell ^-\ell ^+\ell ^-$ analysis, for both the ggF and VBF production modes and resonance masses of 300 and 600 $\text {GeV}$. The acceptance is defined as the ratio of the number of reconstructed events after all selection requirements to the number of simulated events for each channel/category

Mass	Production mode	ggF-enriched categories			VBF-enriched category (%)
		$4\mu$ channel (%)	$2e2\mu$ channel (%)	4*e* channel (%)	
300 $\text {GeV}$	ggF	56	48	40	1
	VBF	36	30	24	21
600 $\text {GeV}$	ggF	64	56	48	3
	VBF	36	34	32	26

Selected quadruplets are required to have their leptons separated from each other by $\Delta R>0.1$ if they are of the same flavour and by $\Delta R>0.2$ otherwise. For $4\mu$ and 4*e* quadruplets, if an opposite-charge same-flavour lepton pair is found with $m_{\ell \ell }$ below 5 $\text {GeV}$, the quadruplet is removed to suppress the contamination from $J/\psi$ mesons. If multiple quadruplets from different channels are selected at this point, only the quadruplet from the channel with the highest expected signal rate is retained, in the order: $4\mu$, $2e2\mu$, 4*e*.

The $Z$ + jets and $t\bar{t}$ background contributions are reduced by imposing impact-parameter requirements as well as track- and calorimeter-based isolation requirements on the leptons. The transverse impact-parameter significance, defined as the impact parameter calculated with respect to the measured beam line position in the transverse plane divided by its uncertainty, $|d_0|/\sigma _{d_0}$, for all muons (electrons) is required to be lower than 3 (5). The normalised track-isolation discriminant, defined as the sum of the transverse momenta of tracks, inside a cone of size $\Delta R=0.3\ (0.2)$ around the muon (electron) candidate, excluding the lepton track, divided by the lepton $p_{\text {T}}$, is required to be smaller than 0.15. The larger muon cone size corresponds to that used by the muon trigger. Contributions from pile-up are suppressed by requiring tracks in the cone to originate from the primary vertex. To retain efficiency at higher $p_{\text {T}}$, the track-isolation cone size is reduced to 10 $\text {GeV}$/$p_{\text {T}}$ for $p_{\text {T}}$ above 33 (50) $\text {GeV}$ for muons (electrons).

The relative calorimetric isolation is computed as the sum of the cluster transverse energies $E_{\text {T}}$, in the electromagnetic and hadronic calorimeters, with a reconstructed barycentre inside a cone of size $\Delta R=0.2$ around the candidate lepton, divided by the lepton $p_{\text {T}}$. The clusters used for the isolation are the same as those for reconstructing jets. The relative calorimetric isolation is required to be smaller than 0.3 (0.2) for muons (electrons). The measured calorimeter energy around the muon (inside a cone of size $\Delta R=0.1$) and the cells within $0.125 \times 0.175$ in $\eta \times \phi$ around the electron barycentre are excluded from the respective sums. The pile-up and underlying-event contributions to the calorimeter isolation are subtracted event by event [71]. For both the track- and calorimeter-based isolation requirements, any contribution arising from other leptons of the quadruplet is subtracted.

An additional requirement based on a vertex-reconstruction algorithm, which fits the four-lepton candidates with the constraint that they originate from a common vertex, is applied in order to further reduce the $Z+\mathrm {jets}$ and $t\bar{t}$ background contributions. A loose cut of $\chi ^{2} / \mathrm {ndof} < 6$ for $4\mu$ and $<9$ for the other channels is applied, which retains a signal efficiency larger than $99 \%$ in all channels.

The QED process of radiative photon production in *Z* boson decays is well modelled by simulation. Some of the final-state-radiation (FSR) photons can be identified in the calorimeter and incorporated into the $\ell ^+\ell ^-\ell ^+\ell ^-$ analysis. The strategy to include FSR photons into the reconstruction of *Z* bosons is the same as in Run 1 [21]. It consists of a search for collinear (for muons) and non-collinear FSR photons (for muons and electrons) with only one FSR photon allowed per event. After the FSR correction, the lepton four-momenta of both dilepton pairs are recomputed by means of a *Z*-mass-constrained kinematic fit. The fit uses a Breit–Wigner *Z* boson line-shape and a single Gaussian function per lepton to model the momentum response function with the Gaussian width set to the expected resolution for each lepton. The *Z*-mass constraint is applied to both *Z* candidates, and improves the $m_{4\ell }$ resolution by about 15%.

In order to be sensitive to the VBF production mode, events are classified into four categories: one for the VBF production mode and three for the ggF production mode, one for each of the three channels. If an event has two or more jets with $p_{\text {T}}$ greater than 30 $\text {GeV}$, with the two leading jets being well separated in $\eta$, $|\Delta \eta _{\text {jj}}| > 3.3$, and having an invariant mass $m_{\text {jj}} > 400~\text {GeV}$, this event is classified into the VBF-enriched category; otherwise the event is classified into one of the ggF-enriched categories. Such classification is used only in the search for a heavy scalar produced with the NWA.

The signal acceptance, defined as the ratio of the number of reconstructed events passing the analysis requirements to the number of simulated events in each category, is shown in Table 1, for the ggF and VBF production modes as well as different resonance masses. The contribution from final states with $\tau$ leptons decaying into electrons or muons is found to be negligible.

**Fig. 1:**
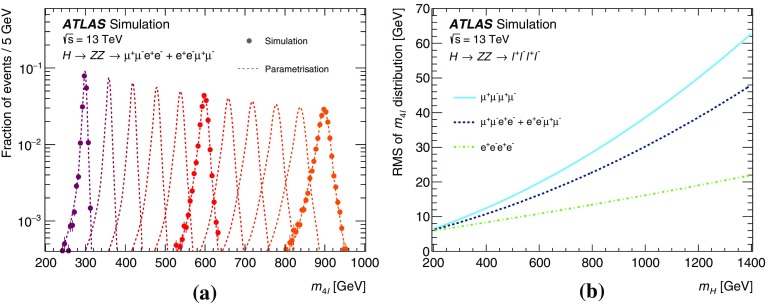
**a** Parameterisation of the four-lepton invariant mass ($m_{4\ell }$) spectrum for various resonance mass ($m_{H}$) hypotheses in the NWA. Markers show the simulated $m_{4\ell }$ distribution for three specific values of $m_{H}$ (300, 600, 900 $\text {GeV}$), normalised to unit area, and the dashed lines show the parameterisation used in the $2e2\mu$ channel for these mass points as well as for intervening ones. **b** RMS of the four-lepton invariant mass distribution as a function of $m_{H}$

### Background estimation

The main background component in the $H\!\rightarrow \!ZZ\!\rightarrow \!\ell ^+\ell ^-\ell ^+\ell ^-$ final state, accounting for 97% of the total expected background events, is non-resonant $ZZ$ production. This arises from quark–antiquark annihilation (86%), gluon-initiated production (10%) and a small contribution from EW vector-boson scattering (1%). The last is more important in the VBF-enriched category, where it accounts for 16% of the total expected background. These backgrounds are all modelled by MC simulation as described in Sect. 3. Additional background comes from the $Z$ + jets and $t\bar{t}$ processes, which contribute at the percent level and decrease more rapidly than the non-resonant $ZZ$ production as a function of $m_{4\ell }$. These backgrounds are estimated using data where possible, following slightly different approaches for final states with a dimuon ($\ell \ell +\mu \mu$) or a dielectron ($\ell \ell +ee$) subleading pair [72].

The $\ell \ell +\mu \mu$ non-$ZZ$ background comprises mostly $t\bar{t}$ and $Z$ + jets events, where in the latter case the muons arise mostly from heavy-flavour semileptonic decays and to a lesser extent from $\pi$/*K* in-flight decays. The contribution from single-top production is negligible. The normalisations of the $Z$ + jets and $t\bar{t}$ backgrounds are determined using fits to the invariant mass of the leading lepton pair in dedicated data control regions. The control regions are formed by relaxing the $\chi ^2$ requirement on the vertex fit, and by inverting and relaxing isolation and/or impact-parameter requirements on the subleading muon pair. An additional control region ($e\mu \mu \mu$) is used to improve the $t\bar{t}$ background estimate. Transfer factors to extrapolate from the control regions to the signal region are obtained separately for $t\bar{t}$ and $Z$ + jets using simulated events. The transfer factors have a negligible impact on the $m_{4\ell }$ shape of the $\ell \ell +\mu \mu$ background.

The main background for the $\ell \ell +ee$ process arises from the misidentification of light-flavour jets as electrons, photon conversions and the semileptonic decays of heavy-flavour hadrons. The $\ell \ell +ee$ control-region selection requires the electrons in the subleading lepton pair to have the same charge, and relaxes the identification and isolation requirements on the electron candidate, denoted *X*, with the lower transverse momentum. The heavy-flavour background is completely determined from simulation, whereas the light-flavour and photon-conversion background is obtained with the sPlot [73] method, based on a fit to the number of hits in the innermost ID layer in the data control region. Transfer factors for the light-flavour jets and converted photons, obtained from simulated samples, are corrected using a $Z+X$ control region and then used to extrapolate the extracted yields to the signal region. Both the yield extraction and the extrapolation are performed in bins of the transverse momentum of the electron candidate and the jet multiplicity.

The *WZ* production process is included in the data-driven estimates for the $\ell \ell +ee$ final states, while it is added from simulation for the $\ell \ell +\mu \mu$ final states. The contributions from $t\bar{t} V$ (where *V* stands for either a *W* or a *Z* boson) and triboson processes are minor and taken from simulated samples.

### Signal and background modelling

The parameterisation of the reconstructed four-lepton invariant mass $m_{4\ell }$ distribution for signal and background is based on the MC simulation and used to fit the data.

In the case of a narrow resonance, the width in $m_{4\ell }$ is determined by the detector resolution, which is modelled by the sum of a Crystal Ball ($\mathcal {C}$) function [74, 75] and a Gaussian ($\mathcal {G}$) function:

$$\begin{aligned} P_{s} (m_{4\ell })= & {} f_{\mathcal {C}} \times \mathcal {C}(m_{4\ell }; \mu , \sigma _{\mathcal {C}}, \alpha _{\mathcal {C}}, n_{\mathcal {C}})\\&+ (1 - f_{\mathcal {C}}) \times \mathcal {G}(m_{4\ell }; \mu , \sigma _{\mathcal {G}}). \end{aligned}$$

The Crystal Ball and the Gaussian functions share the same peak value of $m_{4\ell }$ ($\mu$), but have different resolution parameters, $\sigma _{\mathcal {C}}$ and $\sigma _{\mathcal {G}}$. The $\alpha _{\mathcal {C}}$ and $n_{\mathcal {C}}$ parameters control the shape and position of the non-Gaussian tail and the parameter $f_{\mathcal {C}}$ ensures the relative normalisation of the two probability density functions. To improve the stability of the parameterisation in the full mass range considered, the parameter $n_{\mathcal {C}}$ is set to a fixed value. The bias in the extraction of signal yields introduced by using the analytical function is below 1.5%. The function parameters are determined separately for each final state using signal simulation, and fitted to first- and second-degree polynomials in scalar mass $m_{H}$ to interpolate between the generated mass points. The use of this parameterisation for the function parameters introduces an extra bias in the signal yield and $m_{H}$ extraction of about 1%. An example of this parameterisation is illustrated in Fig. 1, where the left plot shows the mass distribution for simulated samples at $m_{H}$ $=300,600,900$ $\text {GeV}$ and the right plot shows the RMS of the $m_{4\ell }$ distribution in the range considered for this search.

In the case of the LWA, the particle-level line-shape of $m_{4\ell }$ is derived from a theoretical calculation, as described in Ref. [76], and is then convolved with the detector resolution, using the same procedure as for the modelling of the narrow resonance.

The $m_{4\ell }$ distribution for the $ZZ$ continuum background is taken from MC simulation, and parameterised by an empirical function for both the quark- and gluon-initiated processes:

$$\begin{aligned} f_{qqZZ/ggZZ}(m_{4\ell })= & {} (f_{1} (m_{4\ell })+ f_{2} (m_{4\ell })) \times H(m_{0}-m_{4\ell })\\&\times C_0 + f_{3} (m_{4\ell }) \times H(m_{4\ell }-m_{0}), \end{aligned}$$

where:

$$\begin{aligned} f_{1} (m_{4\ell })&= \exp (a_1 + a_2 \cdot m_{4\ell }), \\ f_{2} (m_{4\ell })&= \left\{ \frac{1}{2} + \frac{1}{2}\,\text {erf}\left( \frac{m_{4\ell }-b_1}{b_2}\right) \right\} \times \frac{1}{1+\exp \left( \frac{m_{4\ell }-b_1}{b_3}\right) }, \\ f_{3} (m_{4\ell })&= \exp (c_1 + c_2 \cdot m_{4\ell } + c_3 \cdot m_{4\ell }^2 + c_4 \cdot m_{4\ell }^{2.7}), \\ C_0&= \frac{f_{3} (m_0)}{f_{1} (m_0)+f_{2} (m_0)}. \end{aligned}$$

The function’s first part, $f_1$, covers the low-mass part of the spectrum where one of the *Z* bosons is off-shell, while $f_2$ models the $ZZ$ threshold around 2$\cdot m_Z$ and $f_3$ describes the high-mass tail. The transition between low- and high-mass parts is performed by the Heaviside step function *H*(*x*) around $m_0 = 240$ $\text {GeV}$. The continuity of the function around $m_0$ is ensured by the normalisation factor $C_0$ that is applied to the low-mass part. Finally, $a_i$, $b_i$ and $c_i$ are shape parameters which are obtained by fitting the $m_{4\ell }$ distribution in simulation for each category. The uncertainties in the values of these parameters from the fit are found to be negligible. The MC statistical uncertainties in the high-mass tail are taken into account by assigning a 1% uncertainty to $c_4$.

The $m_{4\ell }$ shapes are extracted from simulation for most background components ($t\bar{t} V$, $VVV$, $\ell \ell +\mu \mu$ and heavy-flavour hadron component of $\ell \ell +ee$), except for the light-flavour jets and photon conversions in the case of $\ell \ell +ee$ background, which is taken from the control region as described in Sect. 5.2.

#### Interference modelling

The gluon-initiated production of a heavy scalar $H$, the SM $h$ and the $gg \rightarrow ZZ$ continuum background all share the same initial and final state, and thus lead to interference terms in the total amplitude. Theoretical calculations described in Ref. [77] have shown that the effect of interference could modify the integrated cross section by up to $\mathcal {O}$(10%), and this effect is enhanced as the width of the heavy scalar increases. Therefore, a search for a heavy scalar Higgs boson in the LWA case must properly account for two interference effects: the interference between the heavy scalar and the SM Higgs boson (denoted by $H$–$h$) and between the heavy scalar and the $gg \rightarrow ZZ$ continuum (denoted by $H$–$B$).

Assuming that $H$ and $h$ bosons have similar properties, as postulated by the 2HDM, they have the same production and decay amplitudes and therefore the only difference between the signal and interference terms in the production cross section comes from the propagator. Hence, the acceptance and resolution of the signal and interference terms are expected to be the same. The $H$–$h$ interference is obtained by reweighting the particle-level line-shape of generated signal events using the following formula:

$$\begin{aligned} w(m_{4\ell }) = \frac{2 \cdot Re \left[ \frac{1}{s-s_H} \cdot \frac{1}{(s-s_h)^*} \right] }{\frac{1}{\left| s - s_H \right| ^2}}, \end{aligned}$$

where $1/\left( s-s_{H (h)}\right)$ is the propagator for a scalar ($H$ or $h$). The particle-level line-shape is then convolved with the detector resolution function, and the signal and interference acceptances are assumed to be the same.

In order to extract the $H$–$B$ interference contribution, signal-only and background-only samples are subtracted from the generated *SBI* samples. The extracted particle-level $m_{4\ell }$ distribution for the $H$–$B$ interference term is then convolved with the detector resolution.

**Fig. 2:**
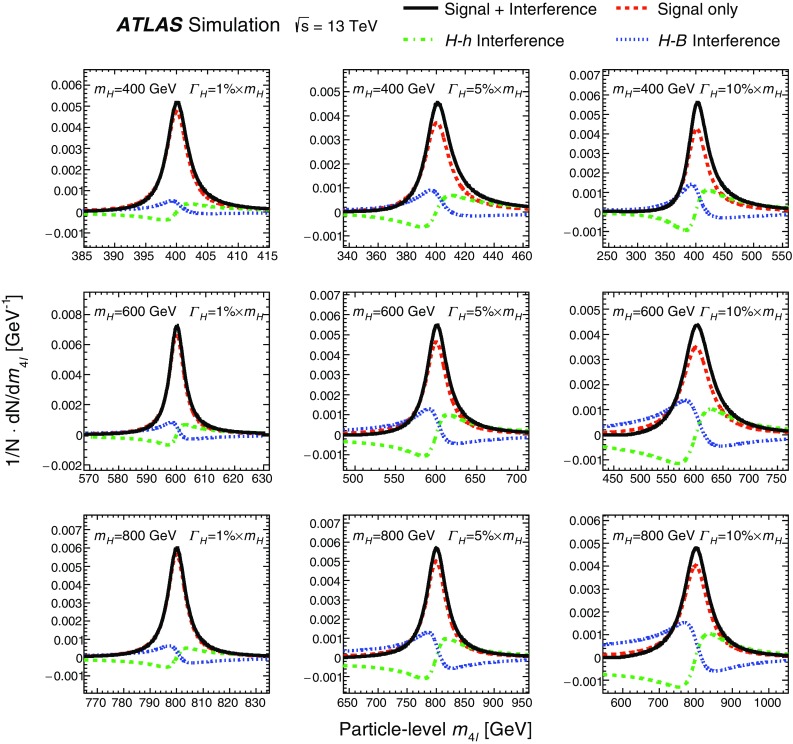
Particle-level four-lepton mass $m_{4\ell }$ model for signal only (red), $H$–$h$ interference (green), $H$–$B$ interference (blue) and the sum of the three processes (black). Three values of the resonance mass $m_{H}$ (400, 600, 800 $\text {GeV}$) are chosen, as well as three values of the resonance width $\Gamma _{H}$ (1, 5, $10\%$ of $m_{H}$). The signal cross section, which determines the relative contribution of the signal and interference, is taken to be the cross section of the expected limit for each combination of $m_{H}$ and $\Gamma _{H}$. The full model (black) is finally normalised to unity and the other contributions are scaled accordingly

Figure 2 shows the overlay of the signal, both interference effects and the total line-shape for different mass and width hypotheses assuming the couplings expected in the SM for a heavy Higgs boson. As can be seen, the two interference effects tend to cancel out, and the total interference yield is for the most part positive, enhancing the signal.

**Table 2:** Signal acceptance for the $\ell ^+\ell ^-\nu \bar{\nu }$ analysis, for both the ggF and VBF production modes and resonance masses of 300 and 600 $\text {GeV}$. The acceptance is defined as the ratio of the number of reconstructed events after all selection requirements to the number of simulated events for each channel/category

Mass	Production mode	ggF-enriched categories		VBF-enriched category (%)
		$\mu ^+\mu ^-$ channel (%)	$e^{+} e^{-}$ channel (%)	
300 $\text {GeV}$	ggF	6	5	$<0.05$
	VBF	2.6	2.4	0.7
600 $\text {GeV}$	ggF	44	44	1
	VBF	27	27	13

## $H\!\rightarrow \!ZZ\!\rightarrow \!\ell ^+\ell ^-\nu \bar{\nu }$ event selection and background estimation

### Event selection

The analysis is designed to select $ZZ\!\rightarrow \!\ell ^+\ell ^-\nu \bar{\nu }$ events (with $\ell = e,\mu$), where the missing neutrinos are identified by a large $E_{\text {T}}^{\text {miss}}$, and to discriminate against the large $Z$ + jets, $WZ$ and top-quark backgrounds.

Events are required to pass either a single-electron or a single-muon trigger, where different $p_{\text {T}}$ thresholds are used depending on the instantaneous luminosity of the LHC. For the 2015 data the electron and muon triggers had $p_{\text {T}}$ thresholds of 24 and 20 $\text {GeV}$ respectively, while for 2016 the muon trigger threshold was increased to 24 $\text {GeV}$. For both triggers, the threshold is set to 26 $\text {GeV}$ when the instantaneous luminosity exceeds the value of $10^{34}$ $\text {cm}^{-2}\text {s}^{-1}$. The trigger efficiency for signal events passing the final selection is about 99%.

Events are selected if they contain exactly two opposite-charge leptons of the same flavour and “medium” identification, with the more energetic lepton having $p_{\text {T}} > 30$ $\text {GeV}$ and the other one having $p_{\text {T}} > 20$ $\text {GeV}$. The same impact-parameter significance criteria as defined in Sect. 5.1 are applied to the selected leptons. Track- and calorimeter-based isolation criteria as defined in Sect. 5.1 are also applied to the leptons, but in this analysis the isolation criteria are optimised by adjusting the isolation threshold so that their selection efficiency is 99%. If an additional lepton with $p_{\text {T}} > 7$ $\text {GeV}$ and “loose” identification is found, the event is rejected to reduce the amount of $WZ$ background. In order to select leptons originating from the decay of a $Z$ boson, the invariant mass of the pair is required to be in the range 76 to 106 $\text {GeV}$. Moreover, since a $Z$ boson originating from the decay of a high-mass particle is boosted, the two leptons are required to be produced with an angular separation of $\Delta R_{\ell \ell }<1.8$.

Events with neutrinos in the final state are selected by requiring $E_{\text {T}}^{\text {miss}} > 120$ $\text {GeV}$, and this requirement heavily reduces the amount of $Z$ + jets background. In signal events with no initial- or final-state radiation the visible $Z$ boson’s transverse momentum is expected to be opposite the missing transverse momentum, and this characteristic is used to further suppress the $Z$ + jets background. The azimuthal angle between the dilepton system and the missing transverse momentum ($\Delta \phi (\ell \ell ,\vec {E}_{\text {T}}^{\text {miss}})$) is thus required to be greater than 2.7 and the fractional $p_{\text {T}}$ difference, defined as $|p_{\text {T}} ^{\text {miss,jet}} - p_{\text {T}} ^{\ell \ell } |/p_{\text {T}} ^{\ell \ell }$, to be less than 20%, where $p_{\text {T}} ^{\text {miss,jet}} = |\vec {E}_{\text {T}}^{\text {miss}} + \Sigma _{\text {jet}}\vec {p_{\text {T}}}^{\text {jet}}|$.

Additional selection criteria are applied to keep only events with $E_{\text {T}}^{\text {miss}}$ originating from neutrinos rather than detector inefficiencies, poorly reconstructed high-$p_{\text {T}}$ muons or mismeasurements in the hadronic calorimeter. If at least one reconstructed jet has a $p_{\text {T}}$ greater than 100 $\text {GeV}$, the azimuthal angle between the highest-$p_{\text {T}}$ jet and the missing transverse momentum is required to be greater than 0.4. Similarly, if $E_{\text {T}}^{\text {miss}}$ is found to be less than 40% of the scalar sum of the transverse momenta of leptons and jets in the event ($H_{\text {T}}$), the event is rejected. Finally, to reduce the $t\bar{t}$ background, events are rejected whenever a *b*-tagged jet is found.

The sensitivity of the analysis to the VBF production mode is increased by creating a dedicated category of VBF-enriched events. The selection criteria, determined by optimising the expected signal significance using signal and background MC samples, require the presence of at least two jets with $p_{\text {T}} > 30$ $\text {GeV}$  where the two highest-$p_{\text {T}}$ jets are widely separated in $\eta$, $|\Delta \eta _{\text {jj}}| > 4.4$, and have an invariant mass $m_{\text {jj}}$ greater than 550 $\text {GeV}$.

The signal acceptance, defined as the ratio of the number of reconstructed events passing the analysis requirements to the number of simulated events in each category, is shown in Table 2, for the ggF and VBF production modes as well as for different resonance masses. The acceptance increases with mass due to a kinematic threshold determined by the $E_{\text {T}}^{\text {miss}}$ selection criteria. Hence the $\ell ^+\ell ^-\nu \bar{\nu }$ search considers only masses of 300 $\text {GeV}$ and above, where its inclusion improves the combined sensitivity.

### Background estimation

The dominant and irreducible background for this search is non-resonant $ZZ$ production, which accounts for about 60% of the expected background events. The second largest background comes from $WZ$ production ($\sim$ 30%) followed by $Z$ + jets production with poorly reconstructed $E_{\text {T}}^{\text {miss}}$ ($\sim$ 6%). Other sources of background are the $WW$, $t\bar{t}$, $Wt$ and $Z \rightarrow \tau \tau$ processes ($\sim$ 3%). Finally, a small contribution comes from $W$ + jets, $t\bar{t}$, single-top-quark and multi-jet processes, with at least one jet misidentified as an electron or muon, as well as from $t\bar{t} V$/$VVV$ events. In both the ggF- and in the VBF-enriched signal regions, the $ZZ$ background is modelled using MC simulation and normalised using SM predictions, as explained in Sect. 3. The remaining backgrounds are mostly estimated using control samples in data.

**Fig. 3:**
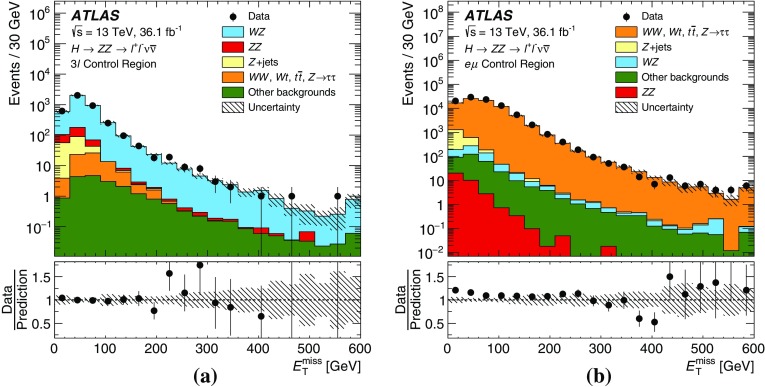
Missing transverse momentum $E_{\text {T}}^{\text {miss}}$ distribution **a** for events in the $3\ell$ control region as defined in the text and **b** for $e^{\pm }\mu ^{\mp }$ lepton pairs after applying the dilepton invariant mass requirement, before applying the rest of the control region selection. The backgrounds are determined following the description in Sect. 6.2 and the last bin includes the overflow. The small excess below 120 $\text {GeV}$ in (**b**) arises from $Z$ + jets background which is here taken from simulation, and lies outside the control region. The error bars on the data points indicate the statistical uncertainty, while the systematic uncertainty in the prediction is shown by the hatched band. The lower panels show the ratio of data to prediction

The $WZ$ background is modelled using simulation but a correction factor for its normalisation is extracted as the ratio of data to simulated events in a dedicated control region, after subtracting from data the non-$WZ$ background contributions. The $WZ$-enriched control sample, called the $3\ell$ control region, is built by selecting $Z \rightarrow \ell \ell$ candidates with an additional electron or muon. This additional lepton is required to satisfy all selection criteria used for the other two leptons, with the only difference that its transverse momentum is required to be greater than 7 $\text {GeV}$. The contamination from $Z$ + jets and $t\bar{t}$ events is reduced by vetoing events with at least one *b*-tagged jet and by requiring the transverse mass of the $W$ boson ($m_{\mathrm {T}}^{W}$), built using the additional lepton and the $E_{\text {T}}^{\text {miss}}$ vector, to be greater than 60 $\text {GeV}$. The distribution of the missing transverse momentum for data and simulated events in the $3\ell$ control region is shown in Fig. 3a. The correction factor derived in the $3\ell$ control region is found to be $1.29\pm 0.09$, where the uncertainty includes effects from the number of events in the control region as well as from experimental systematic uncertainties. Since there are few events after applying all the VBF selection requirements to the $WZ$-enriched control sample, the estimation for the VBF-enriched category is performed by including in the $3\ell$ control region only the requirement of at least two jets with $p_{\text {T}} > 30$ $\text {GeV}$. Finally, a transfer factor is derived from MC simulation by calculating the probability of events satisfying all analysis selection criteria and containing two jets with $p_{\text {T}} > 30$ $\text {GeV}$ to satisfy the $|\Delta \eta _{\text {jj}}| > 4.4$ and $m_{\text {jj}} > 550$ $\text {GeV}$ requirements. An additional systematic uncertainty obtained from the comparison of the $|\Delta \eta _{\text {jj}}|$ distribution between Sherpa and Powheg-Box generators is included to cover potential mismodellings of the VBF selection. Such systematic uncertainty is included in all background estimations when extrapolating from a control region.

The non-resonant background includes mainly $WW$, $t\bar{t}$ and $Wt$ processes, but also $Z \rightarrow \tau \tau$ events in which the $\tau$ leptons produce light leptons and $E_{\text {T}}^{\text {miss}}$. It is estimated by using a control sample of events with lepton pairs of different flavour ($e^{\pm }\mu ^{\mp }$), satisfying all analysis selection criteria.

Figure 3b shows the missing-transverse-momentum distribution for $e^{\pm }\mu ^{\mp }$ events in data and simulation after applying the dilepton invariant-mass selection but before applying the other selection requirements. The non-resonant background in the $e^{+} e^{-}$ and $\mu ^+\mu ^-$ channels is estimated by applying a scale factor (*f*) to the selected events in the $e^{\pm }\mu ^{\mp }$ control region, such that:

$$\begin{aligned} N_{ee}^{\text {bkg}} = \frac{1}{2} \times N_{e\mu }^{\text {data,sub}} \times f, \quad N_{\mu \mu }^{\text {bkg}} = \frac{1}{2} \times N_{e\mu }^{\text {data,sub}} \times \frac{1}{f}, \end{aligned}$$

where $N_{ee}^{\text {bkg}}$ and $N_{\mu \mu }^{\text {bkg}}$ are the numbers of electron- and muon-pair events estimated in the signal region and $N_{e\mu }^{\text {data,sub}}$ is the number of events in the $e^{\pm }\mu ^{\mp }$ control sample with $ZZ$, $WZ$ and other small backgrounds subtracted using simulation. The factor *f* takes into account the different selection efficiencies of $e^{+} e^{-}$ and $\mu ^+\mu ^-$ pairs at the level of the $Z \rightarrow \ell \ell$ selection, and is measured from data as $f^2 = N_{ee}^{\text {data}}/N_{\mu \mu }^{\text {data}}$, where $N_{ee}^{\text {data}}$ and $N_{\mu \mu }^{\text {data}}$ are the numbers of events passing the $Z$ boson mass requirement ($76<m_{\ell \ell }<106$ $\text {GeV}$) in the electron and muon channel respectively. As no events survive in the $e^{\pm }\mu ^{\mp }$ control region after applying the full VBF selection, the background estimation is performed by including only the requirement of at least two jets with $p_{\text {T}} > 30$ $\text {GeV}$. The efficiency of the remaining selection requirements on $|\Delta \eta _{\text {jj}}|$ and $m_{\text {jj}}$ is obtained from simulated events.

The number of $Z$ + jets background events in the signal region is estimated from data, using a so-called ABCD method [78], since events with no genuine $E_{\text {T}}^{\text {miss}}$ in the final state are difficult to model using simulation. The method combines the selection requirements presented in Sect. 6.1 (with $n_{b \text {-tags}}$ representing the number of *b*-tagged jets in the event) into two Boolean discriminants, $V_{1}$ and $V_{2}$, defined as:

$$\begin{aligned} V_{1}\equiv & {} E_{\text {T}}^{\text {miss}}> 120~\text {GeV}\mathrm {and}E_{\text {T}}^{\text {miss}}/ H_{\text {T}}> 0.4, \\ V_{2}\equiv & {} |p_{\text {T}} ^{\text {miss,jet}} - p_{\text {T}} ^{\ell \ell } |/p_{\text {T}} ^{\ell \ell }< 0.2 \mathrm {and}\Delta \phi (\ell \ell ,\vec {E}_{\text {T}}^{\text {miss}})\\&> 2.7 \mathrm {and}\Delta R_{\ell \ell } < 1.8 \mathrm {and}n_{b \text {-tags}} = 0, \end{aligned}$$

with all events required to pass the trigger and dilepton invariant-mass selections. The signal region (A) is thus obtained by requiring both $V_{1}$ and $V_{2}$ to be true, control regions B and C require only one of the two Boolean discriminants to be false ($V_{1}$ and $V_{2}$ respectively) and finally control region D is defined by requiring both $V_{1}$ and $V_{2}$ to be false. With this definition, an estimate of the number of events in region A is given by $N_{\text {A}}^{\text {est}} = N_{\text {C}}^{\text {obs}} \times (N_{\text {B}}^{\text {obs}}/N_{\text {D}}^{\text {obs}})$, where $N_{\text {X}}^{\text {obs}}$ is the number of events observed in region X after subtracting non-$Z$-boson backgrounds. This relation holds as long as the correlation between $V_{1}$ and $V_{2}$ is small, and this is achieved by introducing two additional requirements on control regions B and D, namely $E_{\text {T}}^{\text {miss}}$ > 30 $\text {GeV}$ and $E_{\text {T}}^{\text {miss}}$/ $H_{\text {T}}$ > 0.1. The estimation of the $Z$ + jets background was cross-checked with another approach in which a control region is defined by inverting the analysis selection on $E_{\text {T}}^{\text {miss}}/ H_{\text {T}}$ and then using $Z$ + jets MC simulation to perform the extrapolation to the signal region, yielding results compatible with the ABCD method. Finally, the estimate for the VBF-enriched category is performed by extrapolating the inclusive result obtained with the ABCD method to the VBF signal region, extracting the efficiency of the two-jet, $|\Delta \eta _{\text {jj}}|$ and $m_{\text {jj}}$ selection criteria from $Z$ + jets simulation.

The $W$ + jets and multi-jet background contributions are estimated from data using a so-called fake-factor method [79]. A control region enriched in fake leptons or non-prompt leptons from decays of hadrons is designed by requiring one lepton to pass all analysis requirements (baseline selection) and the other one to not pass either the lepton “medium” identification or the isolation criteria (inverted selection). The background in the signal region is then derived using a transfer factor, measured in a data sample enriched in $Z$ + jets events, as the ratio of jets passing the baseline selection to those passing the inverted selection.

Finally, the background from the $t\bar{t} V$ and $VVV$ processes is estimated using MC simulation.

### Signal and background modelling

The modelling of the transverse mass $m_{\mathrm {T}}$ distribution for signal and background is based on templates derived from fully-simulated events and afterwards used to fit the data. In the case of a narrow resonance, simulated MC events generated for fixed mass hypotheses as described in Sect. 3 are used as the inputs in the moment-morphing technique [80] to obtain the $m_{\mathrm {T}}$ distribution for any other mass hypothesis.

The extraction of the interference terms for the LWA case is performed in the same way as in the $\ell ^+\ell ^-\ell ^+\ell ^-$ final state, as described in Sect. 5.3. In the case of the $\ell ^+\ell ^-\nu \bar{\nu }$ final state a correction factor, extracted as a function of $m_{ZZ}$, is used to reweight the interference distributions obtained at particle level to account for reconstruction effects. The final expected LWA $m_{\mathrm {T}}$ distribution is obtained from the combination of the interference distributions with simulated $m_{\mathrm {T}}$ distributions, which are interpolated between the simulated mass points with a weighting technique using the Higgs propagator, a method similar to that used for the interference.

## Systematic uncertainties

The systematic uncertainties can be classified into experimental and theoretical uncertainties. The first category relates to the reconstruction and identification of leptons and jets, their energy scale and resolution, and the integrated luminosity. Systematic uncertainties in the data-driven background estimates are also included in this category. The second category includes uncertainties in the theoretical description of the signal and background processes.

In both cases the uncertainties are implemented as additional nuisance parameters (NP) that are constrained by a Gaussian distribution in the profile likelihood ratio, as discussed in Sect. 8.1. The uncertainties affect the signal acceptance, its selection efficiency and the discriminant distributions as well as the background estimates for both final states. Each source of uncertainty is either fully correlated or anti-correlated among the different channels and categories.

### Experimental uncertainties

The uncertainty in the combined 2015 and 2016 integrated luminosity is $3.2\%$. This is derived from a preliminary calibration of the luminosity scale using *x*–*y* beam-separation scans performed in August 2015 and May 2016, following a methodology similar to that detailed in Ref. [81].

The lepton identification and reconstruction efficiency and energy/momentum scale and resolution are derived from data using large samples of $J/\psi \rightarrow \ell \ell$ and $Z \rightarrow \ell \ell$ decays. The uncertainties in the reconstruction performance are computed following the method described in Ref. [63] for muons and Ref. [62] for electrons. Typical uncertainties in the identification and reconstruction efficiency are in the range 0.5–3.0% for muons and 1.0%–1.7% for electrons. The uncertainties in the electron energy scale, the muon momentum scale and their resolutions are small, and are fully correlated between the two searches ($\ell ^+\ell ^-\ell ^+\ell ^-$ and $\ell ^+\ell ^-\nu \bar{\nu }$ final states).

The uncertainties in the jet energy scale and resolution have several sources, including uncertainties in the absolute and relative *in situ* calibration, the correction for pile-up, the flavour composition and response [66]. These uncertainties are separated into independent components, which are fully correlated between the two searches. They vary from 4.5% for jets with transverse momentum $p_{\text {T}}$ = 20 $\text {GeV}$, decreasing to 1% for jets with $p_{\text {T}} = 100$–1500 $\text {GeV}$ and increasing again to 3% for jets with higher $p_{\text {T}}$, for the average pile-up conditions of the 2015 and 2016 data-taking period.

Uncertainties in the lepton and jet energy scales are propagated to the uncertainty in the $E_{\text {T}}^{\text {miss}}$. Additionally, the uncertainties from the momentum scale and resolution of the tracks that are not associated with any identified lepton or jet contribute 8 and 3% respectively, to the uncertainty in the $E_{\text {T}}^{\text {miss}}$ value.

The efficiency of the lepton triggers in events with reconstructed leptons is nearly 100%, and hence the related uncertainties are negligible.

### Theoretical uncertainties

For simulated signal and backgrounds, theoretical modelling uncertainties associated with the PDFs, missing QCD higher-order corrections (via variations of factorisation and renormalisation scales), and parton showering are considered.

For all signal hypotheses under consideration, the largest theoretical modelling uncertainties are due to missing QCD higher-order corrections and parton showering. The missing QCD higher-order corrections for ggF production events that fall into the VBF-enriched category are accounted for by varying the scales in MadGraph5_aMC@NLO and affect the signal acceptance by 10%. Parton showering uncertainties are of order 10% and are estimated by comparing Pythia 8.212 to Herwig++ [82].

For the $q\bar{q} \rightarrow ZZ$ background, the effect of the PDF uncertainties in the full mass range varies between 2% and 5% in all categories, and that of missing QCD higher-order corrections is about 10% in the ggF-enriched categories and 30% in the VBF-enriched category. The parton-shower uncertainties result in less than 1% impact in the ggF-enriched categories and about 10% impact in the VBF-enriched category.

For the $gg \rightarrow ZZ$ background, as described in Sect. 3, a 60% relative uncertainty in the inclusive cross section is considered, while a 100% uncertainty is assigned in the VBF-enriched category.

## Results and interpretations

### Statistical procedure

The statistical treatment of the data follows the procedure for the Higgs-boson search combination [83, 84], and is implemented with RooFit [85] and RooStats [86]. The test statistic employed for hypothesis testing and limit setting is the profiled likelihood ratio $\Lambda (\alpha , \varvec{\theta })$, which depends on one or more parameters of interest $\alpha$, and additional nuisance parameters $\varvec{\theta }$. The parameter of interest is the cross section times branching ratio for heavy-resonance production, assumed to be correlated between the two searches. The nuisance parameters represent the estimates of the systematic uncertainties and are each constrained by a Gaussian distribution. For each category of each search, a likelihood fit to the kinematic distribution of a discriminating variable is used to further separate signal from background. The $\ell ^+\ell ^-\ell ^+\ell ^-$ final state uses $m_{4\ell }$ as the discriminant in each category, while the $\ell ^+\ell ^-\nu \bar{\nu }$ final state uses $m_{\mathrm {T}}$ in each category except for the VBF-enriched one where only the overall event counts are used.

As discussed in Sect. 7, the signal acceptance uncertainties, and many of the background theoretical and experimental uncertainties, are treated as fully correlated between the searches. A given correlated uncertainty is modelled in the fit by using a nuisance parameter common to all of the searches. The impact of a systematic uncertainty on the result depends on the production mode and the mass hypothesis. For ggF production, at lower masses the luminosity uncertainty, the modelling uncertainty of the $Z$ + jets background and the statistical uncertainty in the $e\mu$ control region of the $\ell ^+\ell ^-\nu \bar{\nu }$ final state dominate, and at higher masses the uncertainties in the electron-isolation efficiency become important, as also seen in VBF production. For VBF production, the dominant uncertainties come from the theoretical predictions of the $ZZ$ events in the VBF category. Additionally at lower masses, the pile-up reweighting and the jet-energy-resolution uncertainties are also important. Table 3 shows the impact of the leading systematic uncertainties on the predicted signal event yield when the cross section times branching ratio is set to the expected upper limit (shown in Fig. 6), for ggF and VBF production modes. The impact of the uncertainty in the integrated luminosity, 3.2%, enters both in the normalisation of the fitted number of signal events as well as in the background predicted by simulation. This leads to a luminosity uncertainty which varies from 4 to 7% across the mass distribution, depending on the signal-to-background ratio.

**Table 3:** Impact of the leading systematic uncertainties on the predicted signal event yield which is set to the expected upper limit, expressed as a percentage of the yield for the ggF (left) and VBF (right) production modes at $m_{H} = 300$, 600, and $1000~\text {GeV}$

ggF production		VBF production	
Systematic source	Impact [%]	Systematic source	Impact [%]
$$m_{H} =300~\text {GeV}$$			
Luminosity	4	Parton showering	9
$Z$ + jets modelling ($\ell ^+\ell ^-\nu \bar{\nu }$)	3.3	Jet energy scale	4
Parton showering	3.2	Luminosity	4
$e\mu$ statistical uncertainty $\ell ^+\ell ^-\nu \bar{\nu }$	3.2	$q\bar{q} \rightarrow ZZ$ QCD scale (VBF-enriched category)	4
$$m_{H} =600~\text {GeV}$$			
Luminosity	6	Parton showering	6
Pile-up reweighting	5	Pile-up reweighting	6
$Z$ + jets modelling ($\ell ^+\ell ^-\nu \bar{\nu }$)	4	Jet energy scale	6
QCD scale of $q\bar{q} \rightarrow ZZ$	3.1	Luminosity	4
$$m_{H} =1000~\text {GeV}$$			
Luminosity	4	Parton showering	6
QCD scale of $gg \rightarrow ZZ$	2.3	Jet energy scale	5
Jet vertex tagger	1.9	$Z$ + jets modelling ($\ell ^+\ell ^-\nu \bar{\nu }$)	4
$Z$ + jets modelling ($\ell ^+\ell ^-\nu \bar{\nu }$)	1.8	Luminosity	4

### General results

**Fig. 4:**
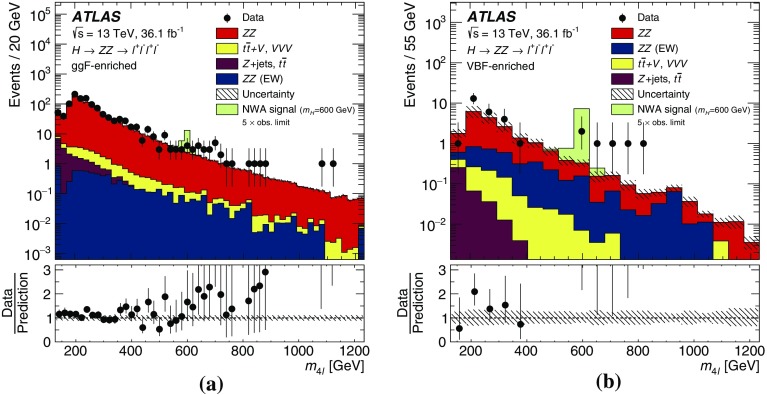
Distribution of the four-lepton invariant mass $m_{4\ell }$ in the $\ell ^+\ell ^-\ell ^+\ell ^-$ search for **a** the ggF-enriched category and **b** the VBF-enriched category. The backgrounds are determined following the description in Sect. 5.2 and the last bin includes the overflow. The simulated $m_{H} = 600$ $\text {GeV}$ signal is normalized to a cross section corresponding to five times the observed limit given in Sect. 8.3.1. The error bars on the data points indicate the statistical uncertainty, while the systematic uncertainty in the prediction is shown by the hatched band. The lower panels show the ratio of data to prediction

The numbers of observed candidate events with mass above 130 $\text {GeV}$ together with the expected background yields are presented in Table 4 for each of the four categories of the $\ell ^+\ell ^-\ell ^+\ell ^-$ analysis. The $m_{4\ell }$ spectrum for the ggF-enriched and VBF-enriched categories is shown in Fig. 4.

**Fig. 5:**
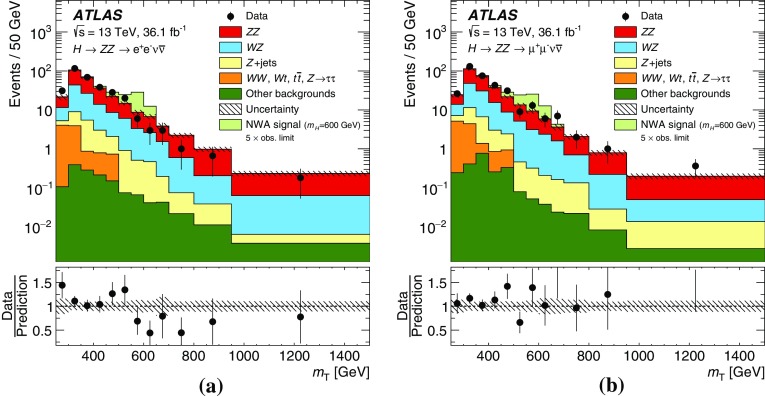
Transverse mass $m_{\mathrm {T}}$ distribution in the $\ell ^+\ell ^-\nu \bar{\nu }$ search for **a** the electron channel and **b** the muon channel, including events from both the ggF-enriched and the VBF-enriched categories. The backgrounds are determined following the description in Sect. 6.2 and the last bin includes the overflow. The simulated $m_{H} = 600$ $\text {GeV}$ signal is normalized to a cross section corresponding to five times the observed limit given in Sect. 8.3.1. The error bars on the data points indicate the statistical uncertainty and markers are drawn at the bin centre. The systematic uncertainty in the prediction is shown by the hatched band. The lower panels show the ratio of data to prediction

Table 5 contains the number of observed candidate events along with the background yields for the $\ell ^+\ell ^-\nu \bar{\nu }$ analysis, while Fig. 5 shows the $m_{\mathrm {T}}$ distribution for the electron and muon channels with the ggF-enriched and VBF-enriched categories combined.

**Table 4:** $\ell ^+\ell ^-\ell ^+\ell ^-$ search: expected and observed numbers of events for $m_{4\ell }$ $> 130$ $\text {GeV}$, together with their statistical and systematic uncertainties, for the ggF- and VBF-enriched categories

Process	ggF-enriched categories			VBF-enriched category
	$4\mu$ channel	$2e2\mu$ channel	4*e* channel	
$ZZ$	$297 \pm 1 \pm 40$	$480 \pm 1 \pm 60$	$193 \pm 1 \pm 25$	$15 \pm 0.1 \pm 6.0$
$ZZ$ (EW)	$1.92 \pm 0.11 \pm 0.19$	$3.36 \pm 0.14 \pm 0.33$	$1.88 \pm 0.12 \pm 0.20$	$3.0 \pm 0.1 \pm 2.2$
$Z$ + jets/$t\bar{t}$/$WZ$	$3.7 \pm 0.1 \pm 0.8$	$7.8 \pm 0.1 \pm 1.1$	$4.4 \pm 0.1 \pm 0.8$	$0.37 \pm 0.01 \pm 0.05$
Other backgrounds	$5.1 \pm 0.1 \pm 0.6$	$8.7 \pm 0.1 \pm 1.0$	$4.0 \pm 0.1 \pm 0.5$	$0.80 \pm 0.02 \pm 0.30$
Total background	$308 \pm 1 \pm 40$	$500 \pm 1 \pm 60$	$203 \pm 1 \pm 25$	$19.5 \pm 0.2 \pm 8.0$
Observed	357	545	256	31

In the $\ell ^+\ell ^-\ell ^+\ell ^-$ search, two excesses are observed in the data for $m_{4\ell }$ around 240 and 700 $\text {GeV}$, each with a local significance of $3.6\sigma$ estimated in the asymptotic approximation, assuming the signal comes only from ggF production. The global significance is $2.2\sigma$ and is calculated, for each excess individually, using the NWA, in the range of 200 $\text {GeV}$< $m_{H}$ < 1200 $\text {GeV}$ using pseudo-experiments.

The excess at 240 $\text {GeV}$ is observed mostly in the 4*e* channel, while the one at 700 $\text {GeV}$ is observed in all channels and categories. No significant deviation from the expected background is observed in the $\ell ^+\ell ^-\nu \bar{\nu }$ final state. The excess observed in the $\ell ^+\ell ^-\ell ^+\ell ^-$ search at a mass around 700 $\text {GeV}$ is excluded at 95% confidence level (CL) by the $\ell ^+\ell ^-\nu \bar{\nu }$ search, which is more sensitive in this mass range. The excess at 240 $\text {GeV}$ is not covered by the $\ell ^+\ell ^-\nu \bar{\nu }$ search, the sensitivity of which starts from 300 $\text {GeV}$. When combining the results from the two final states, the largest deviation with respect to the background expectation is observed around 700 $\text {GeV}$ with a global significance of less than $1\sigma$ and a local significance of about $2\sigma$. The combined yield of the two final states is 1870 events observed in data compared to $1643\pm 164$ (combined statistical and systematic uncertainty) for the expected background. This corresponds to a $1.3\sigma$ global excess in data. Since no significant excess is found, the results are interpreted as upper limits on the production cross section of a spin-0 or spin-2 resonance.

**Table 5:** $\ell ^+\ell ^-\nu \bar{\nu }$ search: expected and observed number of events together with their statistical and systematic uncertainties, for the ggF- and VBF-enriched categories

Process	ggF-enriched categories		VBF-enriched category
	$e^{+} e^{-}$ channel	$\mu ^+\mu ^-$ channel	
$ZZ$	$177 \pm 3 \pm 21$	$180 \pm 3 \pm 21$	$2.1 \pm 0.2 \pm 0.7$
$WZ$	$93 \pm 2 \pm 4$	$99.5 \pm 2.3 \pm 3.2$	$1.29 \pm 0.04 \pm 0.27$
$WW$/$t\bar{t}$/$Wt$/$Z \rightarrow \tau \tau$	$9.2 \pm 2.2 \pm 1.4$	$10.7 \pm 2.5 \pm 0.9$	$0.39 \pm 0.24 \pm 0.26$
$Z$ + jets	$17 \pm 1 \pm 11$	$19 \pm 1 \pm 17$	$0.8 \pm 0.1 \pm 0.5$
Other backgrounds	$1.12 \pm 0.04 \pm 0.08$	$1.03 \pm 0.04 \pm 0.08$	$0.03 \pm 0.01 \pm 0.01$
Total background	$297 \pm 4 \pm 24$	$311 \pm 5 \pm 27$	$4.6 \pm 0.4 \pm 0.9$
Observed	320	352	9

### Spin-0 resonance interpretation

Limits from the combination of the two searches in the context of a spin-0 resonance are described below.

#### NWA interpretation

**Fig. 6:**
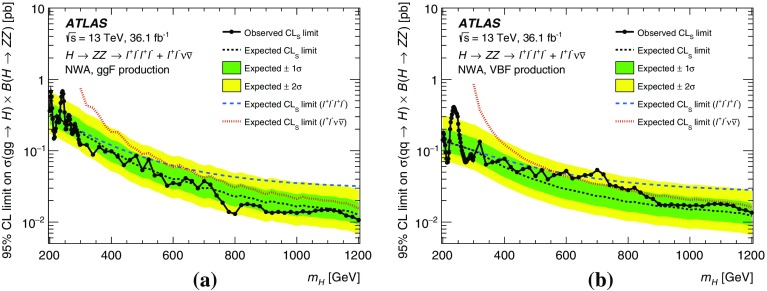
The upper limits at 95% CL on the cross section times branching ratio as a function of the heavy resonance mass $$m_{H} $$ for **a** the ggF production mode($$\sigma _{\text {ggF}} \times B(H\,\rightarrow \,ZZ\,)$$) and **b** for the VBF production mode ($$\sigma _{\text {VBF}} \times B(H\,\rightarrow \,ZZ\,)$$) in the case of the NWA. The green and yellow bands represent the $$\pm $$ $$1\sigma $$ and $$\pm $$ $$2\sigma $$ uncertainties in the expected limits. The dashed coloured lines indicate the expected limits obtained from the individual searches

Upper limits on the cross section times branching ratio ($\sigma \times B(H\,\rightarrow \,ZZ\,)$) for a heavy resonance are obtained as a function of $m_{H}$ with the $\mathrm {CL}_{\mathrm {s}}$ procedure [87] in the asymptotic approximation from the combination of the two final states. It is assumed that an additional heavy scalar would be produced predominantly via the ggF and VBF processes but that the ratio of the two production mechanisms is unknown in the absence of a specific model. For this reason, fits for the ggF and VBF production processes are done separately, and in each case the other process is allowed to float in the fit as an additional nuisance parameter. Figure 6 presents the observed and expected limits at 95% CL on $\sigma \times B(H\,\rightarrow \,ZZ\,)$ of a narrow scalar resonance for the ggF (left) and VBF (right) production modes, as well as the expected limits from the $\ell ^+\ell ^-\ell ^+\ell ^-$ and $\ell ^+\ell ^-\nu \bar{\nu }$ searches. This result is valid for models in which the width is less than 0.5% of $m_{H}$. When combining the two final states, the 95% CL upper limits range from 0.68 pb at $m_{H} = 242$ $\text {GeV}$ to 11 fb at $m_{H} = 1200$ $\text {GeV}$ for the ggF production mode and from 0.41 pb at $m_{H} = 236$ $\text {GeV}$ to 13 fb at $m_{H} = 1200$ $\text {GeV}$ for the vector-boson fusion production mode. Compared with the results from Run 1 [21], where all four final states of $ZZ$ decays were combined, the exclusion region presented here is significantly extended considering that the ratios of parton luminosities [88] increase by factors of about two to seven for heavy scalar masses from 200 $\text {GeV}$ to 1200 $\text {GeV}$.

#### LWA interpretation

**Fig. 7:**
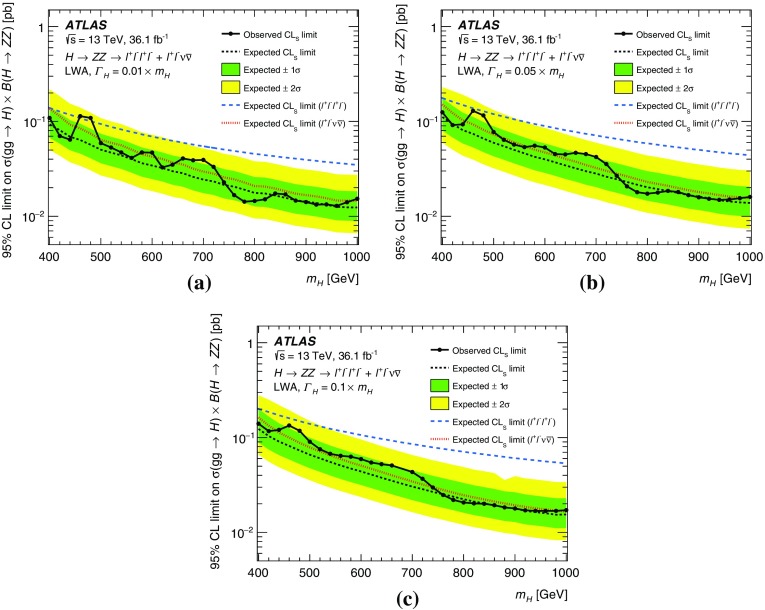
The upper limits at 95% CL on the cross section for the ggF production mode times branching ratio ($\sigma _{\text {ggF}}\times B(H\,\rightarrow \,ZZ\,)$) as function of $m_{H}$ for an additional heavy scalar assuming a width of **a** 1%, **b** 5%, and **c** 10% of $m_{H}$. The green and yellow bands represent the $\pm$ $1\sigma$ and $\pm$ $2\sigma$ uncertainties in the expected limits. The dashed coloured lines indicate the expected limits obtained from the individual searches

In the case of the LWA, limits on the cross section for the ggF production mode times branching ratio ($\sigma _{\text {ggF}} \times B(H\,\rightarrow \,ZZ\,)$) are set for different widths of the heavy scalar. The interference between the heavy scalar and the SM Higgs boson, $H$–$h$, as well as the heavy scalar and the $gg \rightarrow ZZ$ continuum, $H$–$B$, are modelled by either analytical functions or reweighting the signal-only events as explained in Sects. 5.3 and 6.3. Figure 7a–c show the limits for a width of 1, 5 and 10% of $m_{H}$ respectively. The limits are set for masses of $m_{H}$ higher than 400 $\text {GeV}$.

#### 2HDM interpretation

A search in the context of a CP-conserving 2HDM is also presented. This model has five physical Higgs bosons after electroweak symmetry breaking: two CP-even, one CP-odd, and two charged. The model considered here has seven free parameters: the Higgs boson masses, the ratio of the vacuum expectation values of the two doublets ($\tan \beta$), the mixing angle between the CP-even Higgs bosons ($\alpha$), and the potential parameter $m_{12}^2$ that mixes the two Higgs doublets. The two Higgs doublets $\Phi _1$ and $\Phi _2$ can couple to leptons and up- and down-type quarks in several ways. In the Type-I model, $\Phi _2$ couples to all quarks and leptons, whereas for Type-II, $\Phi _1$ couples to down-type quarks and leptons and $\Phi _2$ couples to up-type quarks. The “lepton-specific” model is similar to Type-I except for the fact that the leptons couple to $\Phi _1$, instead of $\Phi _2$; the “flipped” model is similar to Type-II except that the leptons couple to $\Phi _2$, instead of $\Phi _1$. In all these models, the coupling of the heaviest CP-even Higgs boson to vector bosons is proportional to $\cos (\beta -\alpha )$. In the limit $\cos (\beta -\alpha ) \rightarrow 0$, the light CP-even Higgs boson is indistinguishable from a SM Higgs boson with the same mass. In the context of $H\,\rightarrow \,ZZ\,$ decays there is no direct coupling of the Higgs boson to leptons, and so only the Type-I and -II interpretations are presented.

**Fig. 8:**
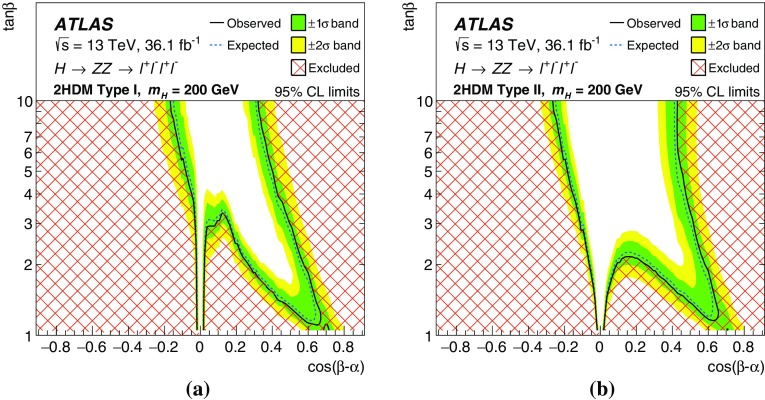
The exclusion contour in the 2HDM **a** Type-I and **b** Type-II models for $m_{H} = 200$ $\text {GeV}$ shown as a function of the parameters $\cos (\beta -\alpha )$ and $\tan \beta$. The green and yellow bands represent the $\pm 1\sigma$ and $\pm 2\sigma$ uncertainties in the expected limits. The hatched area shows the observed exclusion

**Fig. 9:**
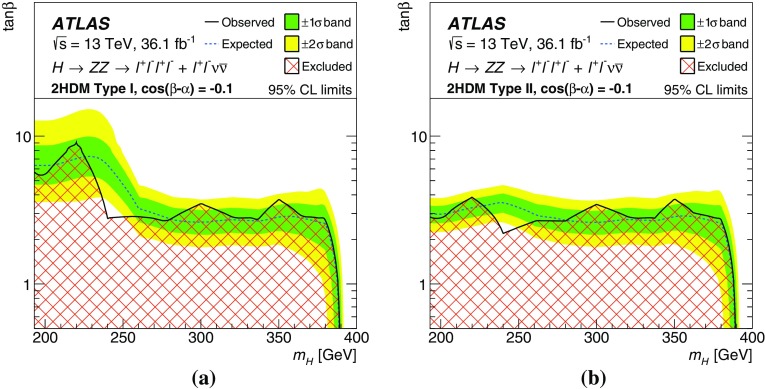
The exclusion contour in the 2HDM **a** Type-I and **b** Type-II models for $\cos (\beta -\alpha )=-0.1$, shown as a function of the heavy scalar mass $m_{H}$ and the parameter $\tan \beta$. The green and yellow bands represent the $\pm 1\sigma$ and $\pm 2\sigma$ uncertainties in the expected limits. The hatched area shows the observed exclusion

Figure 8 shows exclusion limits in the $\tan \beta$ versus $\cos (\beta -\alpha )$ plane for Type-I and Type-II 2HDMs, for a heavy Higgs boson with mass $m_{H}$ = 200 $\text {GeV}$. This $m_{H}$ value is chosen so that the assumption of a narrow Higgs boson is valid over most of the parameter space, and the experimental sensitivity is maximal. At this low mass, only the $\ell ^+\ell ^-\ell ^+\ell ^-$ final state contributes to this result. The range of $\cos (\beta -\alpha )$ and $\tan \beta$ explored is limited to the region where the assumption of a heavy narrow Higgs boson with negligible interference is valid. When calculating the limits at a given choice of $\cos (\beta -\alpha )$ and $\tan \beta$, the relative rates of ggF and VBF production in the fit are set to the prediction of the 2HDM for that parameter choice. Figure 9 shows exclusion limits as a function of the heavy Higgs boson mass $m_{H}$ and the parameter $\tan \beta$ for $\cos (\beta -\alpha ) = -0.1$. The white regions in the exclusion plots indicate regions of parameter space which are not excluded by the present analysis. In these regions the cross section predicted by the 2HDM is below the observed cross section limit. Compared with the results from Run 1 [21], the exclusion presented here is almost twice as stringent.

### Spin-2 resonance interpretation

**Fig. 10:**
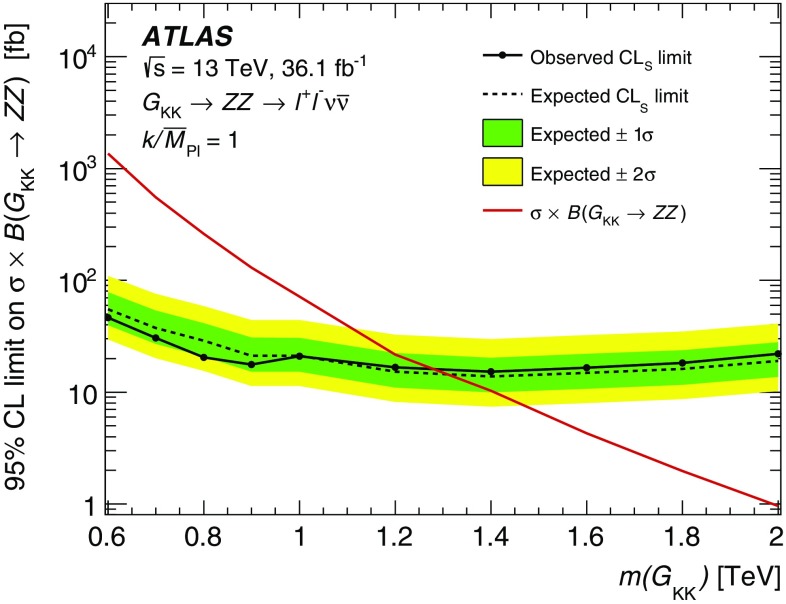
The upper limits at 95% CL on cross section times branching ratio $\sigma \times B(G_{\mathrm {KK}}\rightarrow ZZ )$ for a KK graviton produced with $k/{\bar{M}_\mathrm {Pl}} = 1$. The green and yellow bands give the $\pm$ $1\sigma$ and $\pm$ $2\sigma$ uncertainties in the expected limits. The predicted production cross section times branching ratio as a function of the $G_{\mathrm {KK}}$ mass $m(G_{\mathrm {KK}})$ is shown by the red solid line

The results are also interpreted as a search for a Kaluza–Klein graviton excitation, $G_{\mathrm {KK}}$, in the context of the bulk RS model using the $\ell ^+\ell ^-\nu \bar{\nu }$ final state because the $\ell ^+\ell ^-\ell ^+\ell ^-$ final state was found to have negligible sensitivity for this type of model. The limits on $\sigma \times B(G_{\mathrm {KK}}\rightarrow ZZ )$ at 95% CL as a function of the KK graviton mass, $m(G_{\mathrm {KK}})$, are shown in Fig. 10 together with the predicted $G_{\mathrm {KK}}$ cross section. A spin-2 graviton is excluded up to a mass of 1300 $\text {GeV}$. These limits have been extracted using the asymptotic approximation, and they were verified to be correct within about 4% using pseudo-experiments.

## Summary

A search is conducted for heavy resonances decaying into a pair of $Z$ bosons which subsequently decay into $\ell ^+\ell ^-\ell ^+\ell ^-$ or $\ell ^+\ell ^-\nu \bar{\nu }$ final states. The search uses proton–proton collision data collected with the ATLAS detector during 2015 and 2016 at the Large Hadron Collider at a centre-of-mass energy of 13 $\text {TeV}$ corresponding to an integrated luminosity of 36.1 $\hbox {fb}^{-1}$. The results of the search are interpreted as upper limits on the production cross section of a spin-0 or spin-2 resonance. The mass range of the hypothetical resonances considered is between 200 and 2000 $\text {GeV}$ depending on the final state and the model considered. The spin-0 resonance is assumed to be a heavy scalar, whose dominant production modes are gluon–gluon fusion and vector-boson fusion and it is studied in the narrow-width approximation and with the large-width assumption. In the case of the narrow-width approximation, limits on the production rate of a heavy scalar decaying into two *Z* bosons are set separately for ggF and VBF production modes. Combining the two final states, 95% CL upper limits range from 0.68 pb at $m_{H} = 242$ $\text {GeV}$ to 11 fb at $m_{H} = 1200$ $\text {GeV}$ for the gluon–gluon fusion production mode and from 0.41 pb at $m_{H} = 236$ $\text {GeV}$ to 13 fb at $m_{H} = 1200$ $\text {GeV}$ for the vector-boson fusion production mode. The results are also interpreted in the context of Type-I and Type-II two-Higgs-doublet models, with exclusion contours given in the $\tan \beta$ versus $\cos (\beta -\alpha )$ (for $m_{H} = 200$ $\text {GeV}$) and $\tan \beta$ versus $m_{H}$ planes. This $m_{H}$ value is chosen so that the assumption of a narrow Higgs boson is valid over most of the parameter space and the experimental sensitivity is maximal. The limits on the production rate of a large-width scalar are obtained for widths of 1, 5 and 10% of the mass of the resonance, with the interference between the heavy scalar and the SM Higgs boson as well as the heavy scalar and the $gg \rightarrow ZZ$ continuum taken into account. In the framework of the Randall–Sundrum model with one warped extra dimension a graviton excitation spin-2 resonance with $m(G_{\mathrm {KK}}) < 1300~\text {GeV}$ is excluded at 95% CL.

## Footnotes

1 The ATLAS experiment uses a right-handed coordinate system with its origin at the nominal interaction point (IP) in the centre of the detector and the *z*-axis along the beam pipe. The *x*-axis points from the IP to the centre of the LHC ring, and the *y*-axis points upward. Cylindrical coordinates $(r,\phi )$ are used in the transverse plane, $\phi$ being the azimuthal angle around the *z*-axis. The pseudorapidity is defined in terms of the polar angle $\theta$ as $\eta =-\ln \tan (\theta /2)$.
